# Biomaterials science and surface engineering strategies for dental peri-implantitis management

**DOI:** 10.1186/s40779-024-00532-9

**Published:** 2024-05-13

**Authors:** Ya-Meng Yu, Yu-Pu Lu, Ting Zhang, Yu-Feng Zheng, Yun-Song Liu, Dan-Dan Xia

**Affiliations:** 1 Department of Dental Materials, Peking University School and Hospital of Stomatology, Beijing, 100081 China; 2 National Center for Stomatology & National Clinical Research Center for Oral Diseases & National Engineering Research Center of Oral Biomaterials and Digital Medical Devices, Beijing Key Laboratory of Digital Stomatology & NHC Key Laboratory of Digital Stomatology & NMPA Key Laboratory for Dental Materials, Beijing, 100081 China; 3 School of Materials Science and Engineering, Peking University, Beijing, 100871 China; 4 Department of Prosthodontics, Peking University School and Hospital of Stomatology, Beijing, 100081 China

**Keywords:** Peri-implantitis, Dental implant, Osteogenic property, Antibacterial activity, Anaerobic bacteria

## Abstract

Peri-implantitis is a bacterial infection that causes soft tissue inflammatory lesions and alveolar bone resorption, ultimately resulting in implant failure. Dental implants for clinical use barely have antibacterial properties, and bacterial colonization and biofilm formation on the dental implants are major causes of peri-implantitis. Treatment strategies such as mechanical debridement and antibiotic therapy have been used to remove dental plaque. However, it is particularly important to prevent the occurrence of peri-implantitis rather than treatment. Therefore, the current research spot has focused on improving the antibacterial properties of dental implants, such as the construction of specific micro-nano surface texture, the introduction of diverse functional coatings, or the application of materials with intrinsic antibacterial properties. The aforementioned antibacterial surfaces can be incorporated with bioactive molecules, metallic nanoparticles, or other functional components to further enhance the osteogenic properties and accelerate the healing process. In this review, we summarize the recent developments in biomaterial science and the modification strategies applied to dental implants to inhibit biofilm formation and facilitate bone-implant integration. Furthermore, we summarized the obstacles existing in the process of laboratory research to reach the clinic products, and propose corresponding directions for future developments and research perspectives, so that to provide insights into the rational design and construction of dental implants with the aim to balance antibacterial efficacy, biological safety, and osteogenic property.

## Background

Peri-implantitis is a polymicrobial infection that occurs at the implant site owing to bacterial contamination and poor oral hygiene [1–4]. Dental plaque is one of the main risk factors for peri-implantitis, which causes inflammatory tissue lesions and alveolar bone loss around dental implants [5]. The prevalence rates of peri-implant mucositis and peri-implantitis were about 80% and 28–56% of the subjects, and about 50% and 12–43% of the implants, respectively, after 5–10 years of implantation [6]. Additionally, the occurrence rates of peri-implantitis are 0.16 per patient-year and 0.10 per implant-year, and approximately one-third of the patients and one-fifth of all implants are affected [7]. Therefore, peri-implant disease is considered one of the greatest threats that seriously impair implant success [8, 9]. In the absence of effective interventions, the inflammatory process may gradually destroy the bone surrounding the implant, eventually leading to implant failure [10].

Generally, clinical treatment strategies for peri-implant infection can be categorized into nonsurgical and surgical access therapies. Nonsurgical methods for peri-implantitis mainly involve mechanical debridement (scaling and sandblasting) and adjunctive therapy (chlorhexidine) with the aim to remove dental plaque; surgical treatment of peri-implantitis commonly uses periodontal flap surgery and guided bone regeneration to restore the bone defect [11–15]. Although the above methods have achieved positive therapeutic effects, mechanical debridement may cause damage to the surface properties of the dental implants, and the use of antibiotics may cause bacterial resistance [11, 16, 17]. Besides, the elimination of biofilm only removed inflammatory stimuli in the surrounding microenvironment but did not completely improve the local inflammation in the jaw caused by bacterial infection [18]. Therefore, the management of peri-implantitis aims at removing the bacterial biofilm and rendering the surface and microenvironment to achieve re-osseointegration [19].

At present, titanium (Ti) alloys and zirconia are commonly used as dental implant materials, corresponding products have been widely used in clinics [20, 21]. Besides, polyether-ether-ketone (PEEK) has shown excellent application potential in implant dentistry and is recognized as a promising material to substitute definitive dental implants [22]. However, these materials do not exhibit outstanding antibacterial activity [23–25]. To prevent the occurrence and development of peri-implantitis, the current research focuses on improving the antibacterial properties of dental implants in a variety of manners, including the application of novel biomaterials and surface modification strategies [26, 27]. This review presents an updated overview of the research conducted on biomaterial science and surface engineering strategies applied to dental implants for the prevention and management of peri-implantitis. Many studies have addressed the topographic design and application of coatings with specific antibacterial materials for dental implants [23, 28–31]. Therefore, the current review discusses biomaterial engineering strategies from two functional perspectives — antibacterial and osteogenic, to provide antibacterial protection while accelerating the process of osseointegration and achieving long-term success of implantation.

For collecting information, the PubMed, Web of Science, and Google Scholar electronic databases were searched on June 10, 2023. The keywords applied to the search were as follows: “peri-implantitis,” “dental implant,” “antibacterial,” “osteogenic,” “titanium,” “titanium alloy,” “zirconia” and “polyether-ether-ketone”. These keywords were combined using Boolean logic operators as appropriate. Inclusion and exclusion criteria were employed to assess the relevance of the research content to the topic of the article. The studies included in this paper mainly focused on oral bacteria rather than pathogenic bacteria causing orthopedic infections. However, some studies have used *Staphylococcus aureus* (*S. aureus*) or *Escherichia coli* (*E. coli*) as representatives in their antibacterial experiments. Only articles that specified the use of biomaterials and modification strategies for dental implants were selected.

Herein, we first outline the antibacterial actions involved in the prevention and management of peri-implantitis. Next, we summarize the advancements in dental implant modification strategies aimed at enhancing antibacterial activity and facilitating osseointegration according to the classification of implant materials. Finally, we propose directions for future developments and research perspectives to achieve an optimal balance between antibacterial efficacy and biological activity, with the aim of improving implant success.

## Risk factors and disease characteristics of peri-implantitis

**Fig. 1:**
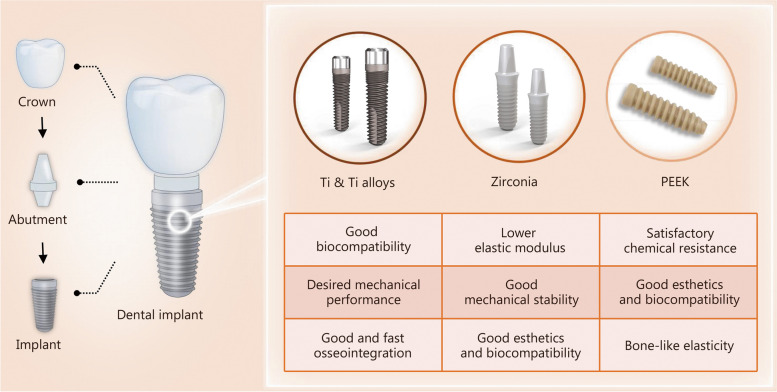
Schematic illustration of structure composition of dental implant and the classification of dental implant biomaterials with their respective advantages. PEEK poly-ether-ether-ketone

For decades, Ti and its alloys and zirconia have been recognized as ideal candidates for dental implants [32] (Fig. 1). PEEK is considered a compelling alternative due to its good physical, chemical, and esthetical properties, though PEEK dental implant has not been commercially available yet [33]. However, the aforementioned materials may not effectively prevent bacterial adhesion [34–36]. The first step in the development of peri-implantitis is the adhesion of free-floating (planktonic) bacteria onto the implant surface, which is affected by several factors, including surface roughness, charge, chemistry, free energy, wettability, and adsorbed proteins [37, 38]. Although bacterial adhesion varies between materials, adhering oral bacteria can proliferate and form robust biofilms that firmly attach to the implant surface, where the bacteria are embedded in a self-produced extracellular polymeric substance and are less susceptible to antibiotics than in their planktonic state [39–41].

The microbiota associated with peri-implantitis is obligate anaerobe Gram-negative bacteria [e.g., *Tannerella forsythia*, *Fusobacterium nucleatum* (*F. nucleatum*), *Treponema denticola*, *Prevotella intermedia*, and *Porphyromonas gingivalis* (*P. gingivalis*)], asaccharolytic anaerobic, Gram-positive rods (e.g., *Eubacterium*) and Gram-positive cocci [e.g., *Streptococcus mutans* and *Streptococcus gordonii* (*S. gordonii*)] [42–45]. Upon implantation, a salivary pellicle is adsorbed onto the orally exposed surfaces, leading to the adhesion of *Streptococcus* and other early colonizers [46]. The early bacterial colonizers provide surface receptors for incremental co-adhesion of secondary colonizers (e.g., *F. nucleatum*) and later colonizers (e.g., *P. gingivalis*) to develop mature biofilm [3]. Studies have confirmed that the core microbiota associated with peri-implantitis (e.g., *Fusobacterium*, *P. gingivalis*, *Eubacterium*, and *Streptococcus*) is similar to that of periodontitis [47–51]. While the pathogen microorganisms isolated from patients with implant-related infections that occur in the long bone are often implicated with *S. aureus* and *E. coli* [52–55]. As peri-implantitis represents a heterogeneous mixed infection that not only includes periodontopathic microorganisms, opportunistic microorganisms such as *E. coli* and *S. aureus* are also found at the infection site [56–58].

**Fig. 2:**
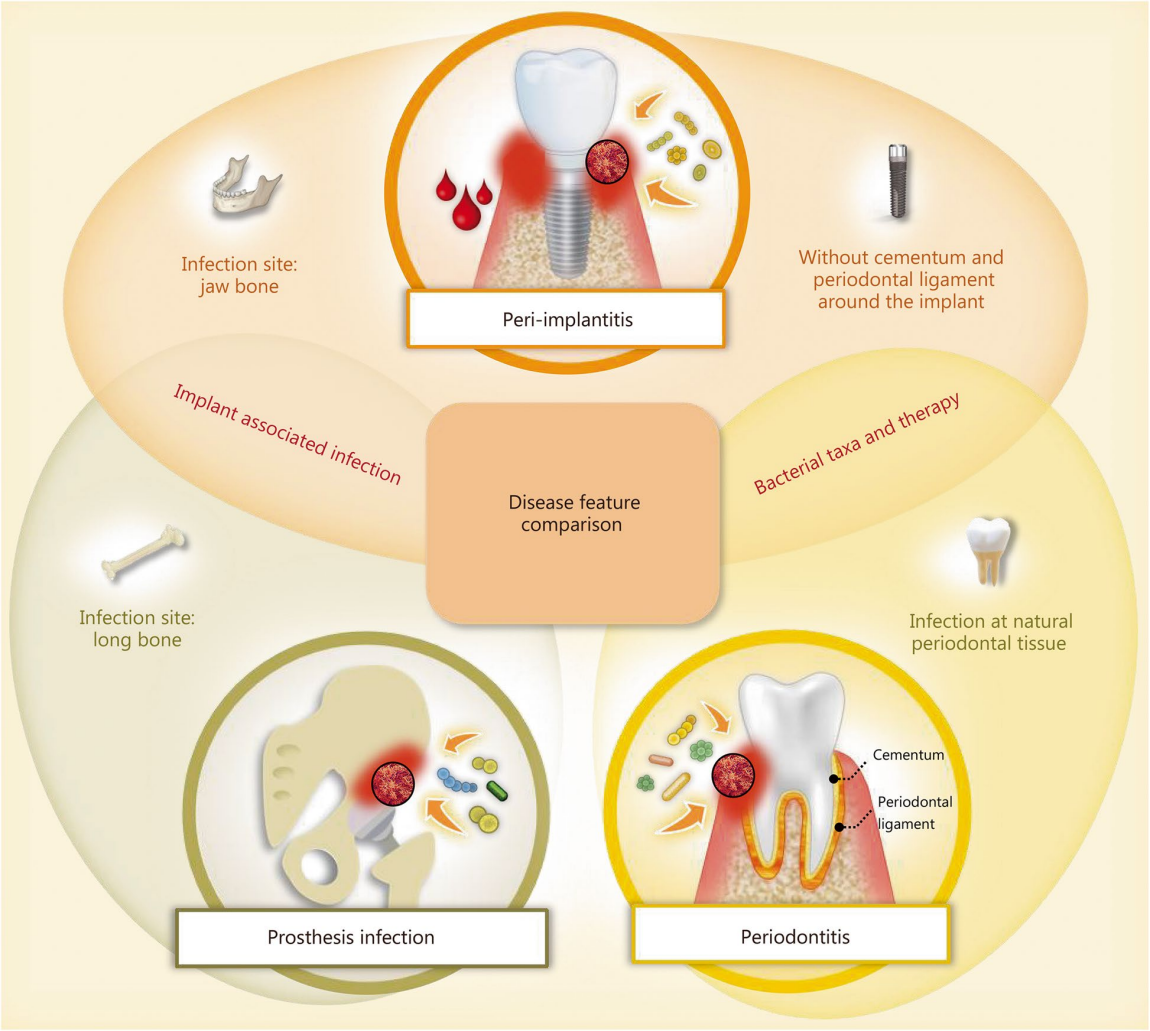
Schematic representation illustrating the distinctions among prosthetic infection, peri-implantitis, and periodontitis in terms of infection site and surrounding tissue structure

Although the clinical symptoms and treatment strategies associated with peri-implantitis are similar to those of periodontitis, they should be recognized as distinct entities (Fig. 2) [59]. Dental implants are more vulnerable to bacterial infections than natural teeth owing to the lack of root cementum and periodontal ligament as protective systems [1, 59]. Furthermore, the microbiome around the implant and the biofilm composition differ from those around teeth, and the management of peri-implantitis is often more difficult and unpredictable compared to periodontitis [60–62]. Therefore, the elimination of bacterial infection is of critical importance in the management of peri-implantitis [4].

## Biological events around the dental implant and “race for the surface theory”

**Fig. 3:**
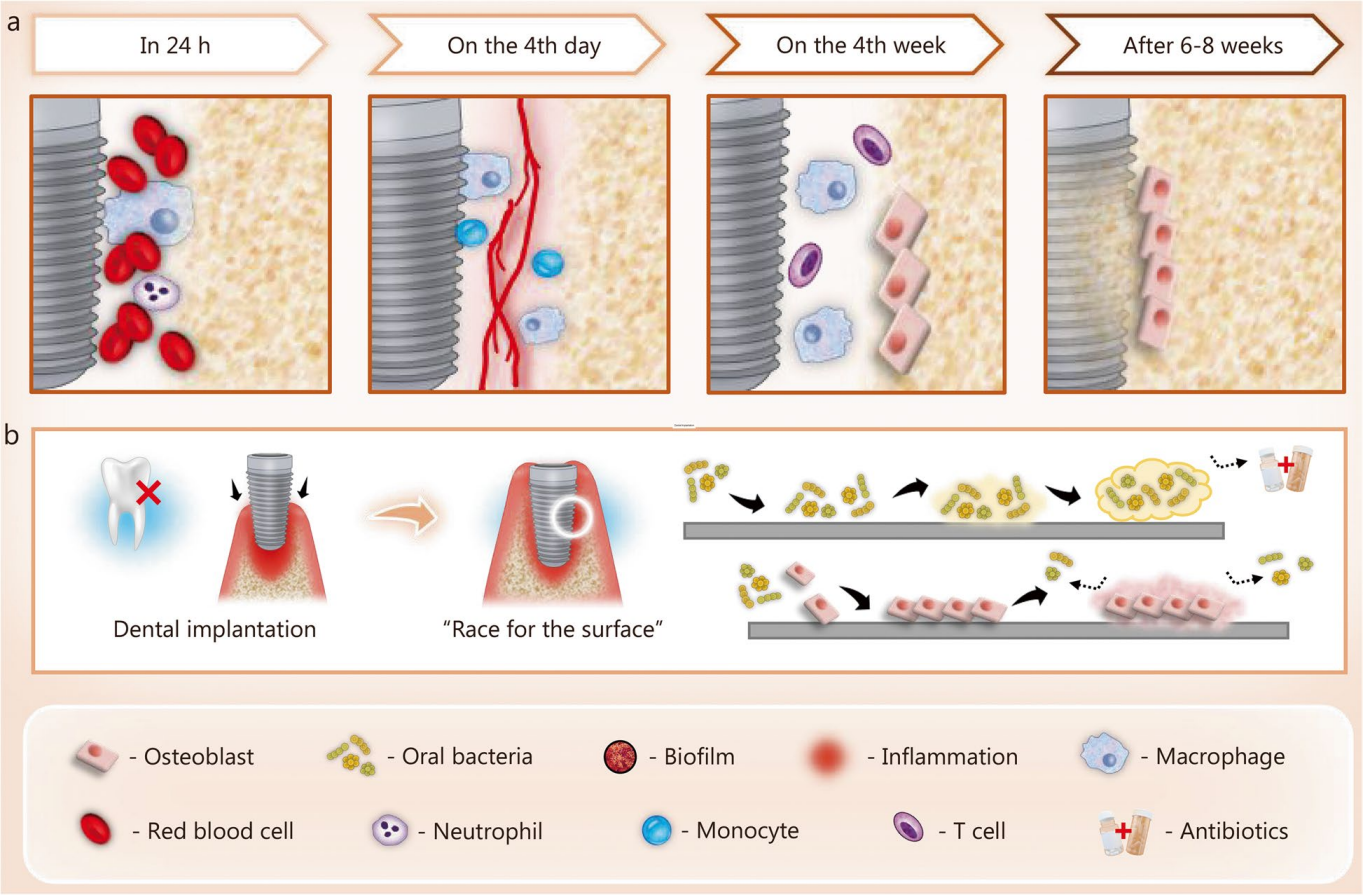
The biological events around dental implants with or without bacterial infection. **a** Biological events at different stages after implantation. **b** “Race for the surface” triggered by bacterial infection following implantation

Bacterial infection-induced peri-implantitis involves a series of complex interactions between pathogens, implant, and the host immune response to both. As shown in Fig. 3a, a list of biological events, including early inflammatory-immuneresponse, neovascularization, and osteogenesis, occur over time after implantation to ensure stable bone-implant integration [12, 63, 64]. However, in bacterial infection-induced peri-implantitis, the pathogenic microorganisms and their sub-products (i.e. lipopolysaccharides) activate innate immune cells (dendritic cells, macrophages, and neutrophils), which are recruited and migrate into the lesion, leading to both innate and acquired immune responses aimed at combating bacterial infections [65]. During this process, macrophages are in an M1 polarization state, resulting in high levels of pro-inflammatory cytokines, especially interleukin (IL)-6 and tumor necrosis factor-α (TNF-α), leading to augmented tissue destruction surrounding the implant [66–68]. Enhancing osteogenic function can not only reduce the adverse impact of inflammatory-immune responses induced by bacterial contamination but also accelerate the healing process and facilitate bone-implant integration. At the moment of insertion, the implant surface can be recognized as a ready substrate for competitive colonization of bacteria and bone tissue cells. This phenomenon is commonly referred to as the “race for the surface” [69] (Fig. 3b). The authors propose that if bone cells emerge as the victors in the competition, it would lead to stable osseointegration, and reduce the implant’s susceptibility to bacterial infection [70]. Therefore, simultaneously improving the antibacterial properties and osteogenic potential of dental implants is of vital significance for the prevention and management of peri-implantitis.

## Clinical treatment strategies for peri-implantitis

Bacterial colonization generally occurs after implantation [71–73]. If no maintenance care, the tissues around the implants will have pathological changes, leading to the formation of dental plaque [74]. Dental plaque formed on the implant surfaces can initiate and promote alveolar bone resorption [75]. According to the 2017 World Workshop on the classification of peri-implant diseases and conditions, the clinical signs of peri-implantitis are characterized by inflammation, bleeding on probing, and/or suppuration, increased probing depths and/or recession of the mucosal margin, as well as radiographic bone loss [76]. The severity of peri-implantitis depends on the probing depth, bleeding, purulence, and extent of bone loss [10, 77]. Once the diagnosis of peri-implantitis has been made, several nonsurgical and surgical treatment strategies are available to eliminate dental plaque and reinstitute osseointegration (Table 1). However, the treatment of peri-implantitis remains controversial, and prevention is recognized as the best way to treat this disease [78].

**Table 1:** Methods of clinical treatment and maintenance for peri-implantitis

Method classification	Treatment method
Nonsurgical methods	Mechanical debridement: manual abrasion, ultrasonic cleaning, sandblasting, laser-blasting, etc.
	Local administration: chlorhexidine, minocycline, doxycycline, metronidazole, etc.
Surgical methods	Periodontal flap surgery: removal of granulation tissue, reducing pathological peri-implant pockets, treatment of the alveolar bone irregularities
	Guided bone regeneration: fill bone defects via autogenous bone, allogeneic bone, or xenograft (used alone or in combination)

## Antibacterial actions involved in the management of bacterial infections

**Fig. 4:**
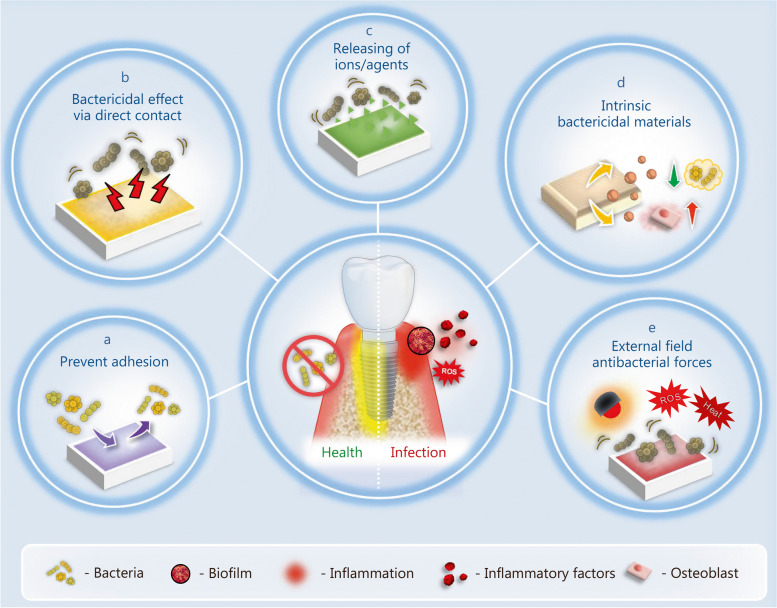
Different antibacterial actions involved in the management of bacterial infections. The antibacterial actions can be divided into 5 categories. **a** Material/surface can inhibit bacterial adhesion. **b** Engineered surfaces can cause bacterial death via direct contact. **c** Engineered surface can release antibacterial ions/agents to achieve bactericidal effects. **d** Material/surface with intrinsic bactericidal effects. **e** Material/surface can be activated by external driving forces to initiate antibacterial activities

Peri-implantitis develops as a result of oral bacteria attachment, proliferation, and formation of mature biofilm [23]. From an antibacterial perspective, protection against bacterial invasion and inhibition of biofilm formation are fundamental strategies for ensuring the long-term success of implants. As shown in Fig. 4, the primary antibacterial actions involved in the management of peri-implant diseases can be categorized into anti-adhesion, bactericidal effects achieved through direct contact or the release of certain ions/agents, and bactericidal actions driven by intrinsic properties of materials or external-field driving forces.

### Prevent bacterial adhesion

Since bacterial adhesion to the surface of dental implants is the first step in the development of biofilms, it is reasonable to endow the surface with anti-adhesion properties to resist bacterial attachment [79]. This can be achieved by grafting hydrophilic polymers, constructing nanoscale topographical patterns, or coating with titanium nitride (TiN) [80–82]. Such surfaces can prevent biofilm formation by inhibiting the attachment of bacteria, rather than providing a bactericidal effect. Additionally, because of their drug-independent antibacterial properties, these surfaces can provide long-term protection and reduce the adverse effects on the surrounding tissue. Among those modification strategies, an in vivo human study was performed to confirm the efficiency of TiN coatings in the prevention of oral bacterial adhesion [83]. However, surfaces with antibacterial attachment properties always show superior anti-fouling performance, which may also hinder the adhesion of bone cells; therefore, bioactive molecules are often used in conjunction with anti-adhesion surfaces to restore the bio-functionality [84]. The other disadvantage is that anti-adhesion surfaces have little influence on species with non-proteinaceous bacterial adhesins [85].

### Bactericidal effect due to direct contact

To effectively provide a bactericidal effect and prevent biofilm development, surfaces are modified with nano-patterns, antimicrobial peptides (AMPs), graphene (G)-based materials, or metal oxide nanoparticles. In nano-patterns surfaces, the presence of sharp protrusions or edges has been observed to effectively destroy bacteria membranes [86, 87]. Similarly, surfaces decorated with AMPs exhibit a mechanism where the positively charged peptides can bind to the negatively charged bacterial cell membrane, leading to disruption of membrane integrity and subsequent activation of autolytic enzymes, thereby preventing drug resistance in bacteria and activating adaptive immunity [88, 89]. Furthermore, surfaces that have been modified with nanoscale G-based materials, metals, metal oxides, nano-sheets, or nano-particles can directly interact with bacterial membranes or trigger the production of reactive oxygen species (ROS) to compromise membrane integrity [90–94]. Numerous in vivo studies have confirmed the bactericidal effects and bone-integration ability of these modifications [93, 95, 96]. While the most frequently used animal models are subcutaneous bacterial infection, bone defect, and heterotopic ossification models, a dental infection model should be established to further verify antibacterial efficiency and osseointegration ability.

### Bactericidal effect due to release of certain ions/agents

Another antimicrobial strategy involves the incorporation of antibacterial drugs, ions, or biomacromolecules into the implants and allowing their active release to provide a bactericidal effect and inhibit biofilm development [97–101]. Various antibacterial agents have been integrated into implant surfaces to provide bactericidal effects through sustained release. The effectiveness of antibacterial drug-loaded coatings largely depends on the coating technique and material used. For example, coatings with highly porous morphology, specific surface area, or multilayered structures may be more beneficial in prolonging the drug release time than those without these properties [99, 102, 103]. Some studies have verified the antibacterial efficacy of such coatings can treat implant-associated infection when exposed to the oral cavity [104, 105]. However, uncontrolled drug release may impair long-term efficiency and cause undesired effects on the surrounding tissues and cells.

### Intrinsic bactericidal actions

Antibacterial alloys are considered to be ideal candidates for dental implants. For instance, Titanium-copper (Ti-Cu) alloys exhibit satisfactory antibacterial efficiency against oral pathogens and reduce biofilm formation [106]. Cu ions released from the bulk material can adversely affect the expression of biofilm-associated genes [107]. Contact sterilization is another effective mechanism of antibacterial activity [108]. Furthermore, Ti-Cu alloys can reduce the stability and structural integrity of biofilm by affecting the production of extracellular polymeric substances and reducing the binding sites for microorganisms [109]. Additionally, Ti-Cu alloys can effectively resist bone resorption caused by bacterial infection while promoting osseointegration [106, 108].

### Bactericidal actions driven from external-field driving forces

Photo-induced bactericidal strategies have drawn increasing attention for their potential to effectively eradicate bacteria without inducing drug resistance, thereby advancing traditional antibiotic methods. The mechanism underlying this photo-induced antibacterial effect involves the production of ROS or hyperthermia [110–112]. Under light irradiation, the generation of ROS (hydroxyl radicals and superoxide anions) or heat can effectively reduce the level of live bacteria and inhibit biofilm development, thereby improving the success rates of dental implants. Near-infrared (NIR) light possesses deep tissue-penetration ability and minimal adsorption of the blood and water molecules in organisms, thus, it is considered to be an ideal external-field force for antibacterial therapy in dental implants [113]. Through the use of the alkaline-acid bidirectional hydrothermal method for surface treatment, a NIR-responsive titanium oxide (TiO_2_)/TiO_2 − X_ super surface was constructed on a Ti-based implant, which exhibited persistent antibacterial activity and effectively alleviated bacteria-induced inflammation in the tissues around implants [111].

## Strategies to enhance Ti-based implant success

Currently, commercially pure (cp.) Ti and Ti alloys (Ti-6Al-4 V) are the most attractive metallic materials for dental implants owing to their good biocompatibility, mechanical performance, and ability to bond with osteoblasts [114, 115]. The surface topography has a significant effect on osseointegration, and enhanced surface roughness can effectively promote bone-implant integration [116–119]. Several modification strategies have been applied to endow Ti-based surfaces with enhanced bone regeneration properties (Table 2) [30, 39, 118, 120–122]. Mainstream modification strategies, including grit blasting and acid etching, are commonly employed to enhance the surface roughness of dental implants [24, 30]. However, the increased surface roughness also provides more surface area for microbial attachment, potentially increasing the risk of bacterial infection [30, 123]. Implants with moderately rough surfaces have been found to accumulate more bacterial biomass and a significantly higher number of oral pathogens than implants with minimally rough surfaces [124].

**Table 2:** Surface modification strategies applied in commercial dental implants

Modification techniques	Commercial products	Surface properties	References
Sandblasting	TiOblast® (Astra Tech, Mölndal, Sweden) Swede and Screw Vent® (Zimmer Biomet, Palm Beach Gardens, Florida, USA) Standard, Hex® (Osteoplant, Poznan, Poland)	Macro-roughness	[30, 120, 121]
Acid etching	Osseotite® (Zimmer Biomet, Warsaw, Indiana, USA) Steri-Oss Etched® (Nobel Biocare, Zürich-Flughafen, Switzerland)	Micro-roughness created by the formation of micro wells on the implant surface	[30, 120, 122]
Grit blasting and acid etching	SLA Straumann® (Straumann Institute, Basel, Switzerland) Ankylos® (Dentsply Friadent, Mannheim, Germany) Friadent Plus® (Dentsply Friadent, Mannheim, Germany) Promote® (Camlog, Basel, Switzerland) Osseonova® (Ziacom, Pinto, Spain)	Hierarchical topography by combination with both macro- and micro-roughness	[30, 39]
Anodization	TiUnite® (Nobel Biocare, Gothenburg, Sweden)	Porous organized surface in the form of TiO_2_ nanotubes	[30, 118]
Plasma spraying	IMZ-TPS® (Dentsply Friadent, Mannhein, Germany) Bonefit® (Straumann Institute, Waldenburg, Switzerland) Restore-TPS® (Lifecore Biomedical, Chaska, Minnesota, USA) Steri-Oss-TPS® (Nobel Biocare, Yorba Linda, California, USA) ITI-TPS® (Straumann Institute, Waldenburg, Germany)	Increased surface hydrophilicity by the formation of a film containing Ti-OH groups	[30, 39, 120]

*SLA* sandblasting and acid etching, *Ti* titanium

**Fig. 5:**
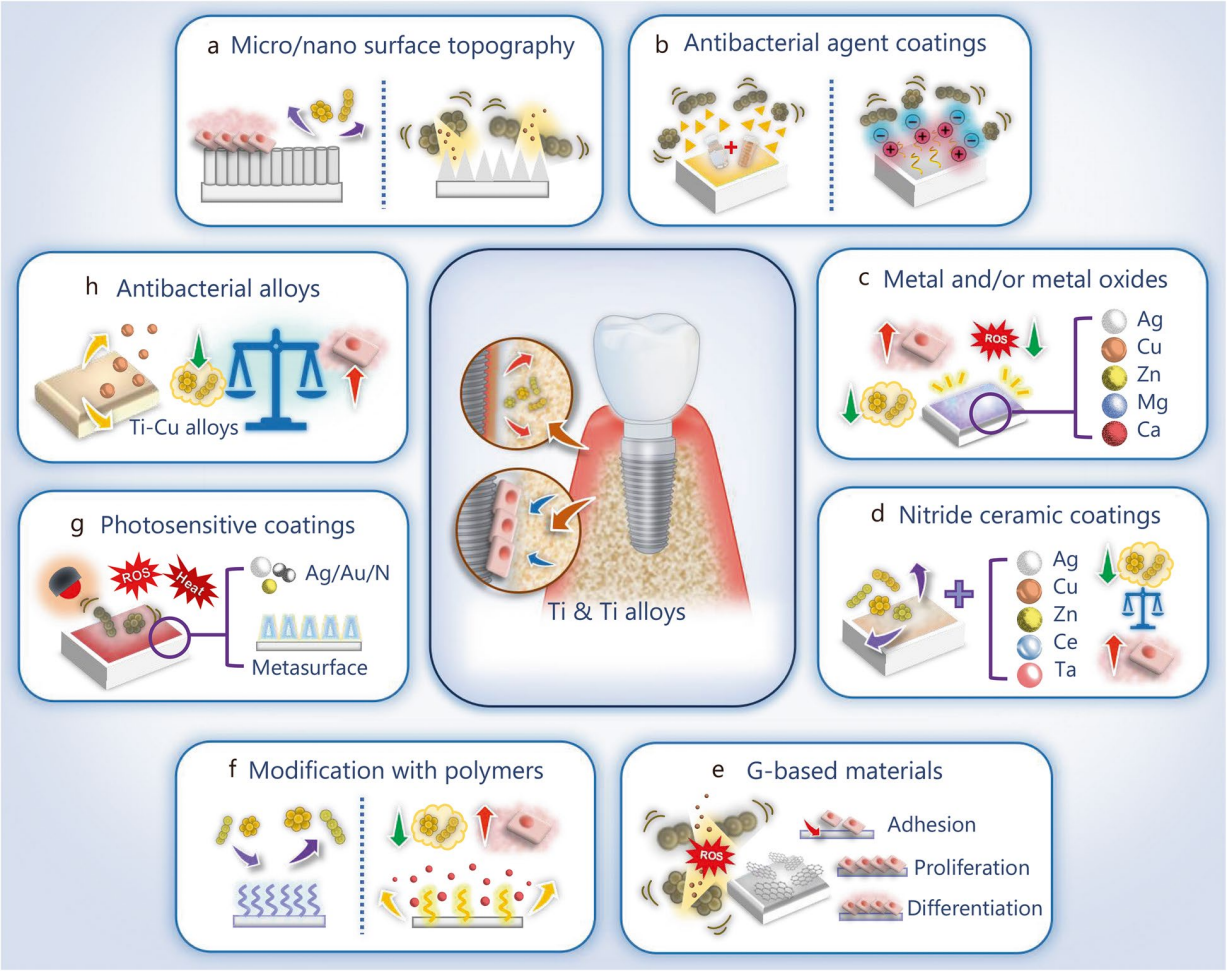
Biomaterials and modification strategies for titanium (Ti)-based dental implants to enhance the osteogenic activities and antibacterial properties. **a** Modification of the surface with micro/nano topography. **b** Coating the surface with an antibacterial agent. **c** Modification the surface with metal and/or metal oxides. **d** Coating the surface with nitride ceramic. **e** Modification of the surface with graphene (G)-based materials. **f** Modification of the surface with functional polymers. **g** Modification of the surface with photosensitive coatings. **h** Application antibacterial alloys. Ag silver, Cu copper, Zn zinc, Ce cerium, Ta tantalum, Mg magnesium, Ca calcium

Ideally, a dental implant should possess satisfactory osseointegration properties while protecting against bacterial infections that cause peri-implant diseases. Therefore, various biomaterials (Fig. 5a-e) and surface-engineering strategies have been utilized to balance antibacterial efficacy, biological safety, and osteogenic properties in order to prevent or manage peri-implantitis (Fig. 5f-h). In the following section, such modification techniques and the related biomaterials used in the process are summarized.

### Micro/nano surface topography with antibacterial efficacy

Surface topographies with varying micro/nano structures exhibit different levels of roughness, wettability, and surface-free energy, resulting in different degrees of antimicrobial properties [125–128]. A commonly used antibacterial surface modification strategy is the introduction of TiO_2_ nanostructure through electrochemical anodization treatment [81, 129]. Furthermore, the combination of microgrooves and nanotubes can promote the attachment and proliferation of osteoblast cells, while reducing the adhesion of *P. gingivalis* [130, 131]. These observations indicate that the proper integration of hierarchical micro/nano patterns on the implant surface can facilitate the selective elimination of bacteria.

In addition to nanotubes, other nano-texture surfaces also showed satisfactory antibacterial effects. Hayles et al. [86] developed antibacterial Ti surfaces by hydrothermal etching to construct sharp spike-like nanostructures that cause mechanical disruption to the attached bacteria. *Streptococcus mutans* (*S. mutans*), *F. nucleatum*, and *P. gingivalis* were chosen as model pathogens to represent the early colonizing bacteria of biofilm formation, pathogens of periodontal diseases, late colonizing bacteria, and keystone pathogens of the oral microbiota. Mukaddam et al. [87] employed helium sputtering to fabricate nanospike-structured surfaces with a spike height of approximately 500 nm. These engineered surfaces effectively prevented the attachment and adhesion of *P. gingivalis* while having minimal effect on gingival fibroblasts.

### Antibacterial agent-loaded surface coatings

#### Antibacterial drugs

Adding antibacterial agents to the surface of dental implants is an effective method to reduce bacterial viability and prevent biofilm development [132]. Doxycycline is a representative antibiotic widely used in dental implants [133]. The doxycycline-coated surface prepared via electrochemical cathodic polarization has long-term bacteriostatic potential, effectively reducing both bacterial growth and biofilm accumulation [134]. Additionally, doxycycline-coated surfaces exhibited enhanced bioactivity both in vitro and in vivo, characterized by increased expression of bone formation-associated genes and increased bone formation markers, without adverse effects on the viability of MC3T3-E1 cells [134]. Therefore, doxycycline is an ideal candidate for dental implant modification. Other antibacterial agents have also been utilized to modify dental implant surfaces, and the types of materials and their preparation methods are listed in Table 3 [97, 133, 135–139]. The effectiveness of such antibacterial coatings depends largely on the coating technique and material used. Coatings with highly porous morphology, large specific surface area, or multilayered structure may be more advantageous for prolonging drug released time. However, some of the surfaces mentioned in the aforementioned studies lacked relevant biocompatibility characterizations. Although totarol has been shown to be mildly cytotoxic, polyhexamethylene biguanide and minocycline are commercially available broad-spectrum antiseptics [140–142]. The optimal load concentration and its influence on the host cells and tissues should be further evaluated in a dose-dependent manner [143–145].

**Table 3:** Summary of the antibacterial agent-loaded coatings

Antibacterial agent		Fabrication method	Bacteria affected	Antibacterial period	Cell response to the surface	In vivo study	References
Biosurfactant	Rhamnolipid	Physical adsorption	*S. aureus*, *S. epidermidis*	3 d	No cytotoxic effect on MRC5 lung fibroblasts	-	[135]
Natural substance	Totarol	Spin coating process	*S. gordonii*, mixed oral bacterial film	2 d	-	-	[97]
Natural antibiotics	Minocycline	Layer-by-layer assembly	*S. aureus*	14 d	-	-	[136]
Antibacterial polymer	PHMB	Hydrogen bonding or physiochemical adsorption	*F. nucleatum*	7 d	-	-	[137]
Antibiotics	Gentamycin	Loading with silica nanoparticles	*S. aureus*	1 d	Biocompatible with primary human skin fibroblasts	-	[138]
Antibiotics	Doxycycline	Electrochemical method	*S. epidermidis*	14 d	No negative effect on MC3T3-E1 cell viability	Rabbit and dog models: enhanced bone formation	[133]
Antibiotics	Vancomycin	Loading with PLGA nanofibers	*S. aureus*	28 d	Enhanced cell viability of MC3T3-E1	Rabbit model: excellent antibacterial performance	[104]
Antibacterial agent	Chlorhexideine gluconate	Internal coating 1% chlorhexideine gluconate	Pathogenic bacteria in the oral cavity	6 months	-	No adverse effect and no implant failure, prevent bacterial infection	[139]

*PHMB* polyhexamethylene biguanide, PLGA poly(lactic-co-glycolic acid), *S. aureus Staphylococcus aureus, S. epidermidis Staphylococcus epidermidis, S. gordonii Streptococcus gordonii, F. nucleatum Fusobacterium nucleatum, MRC5* human embryo lung fibroblasts, *MC3T3-E1* mouse embryonic osteoblast precursor cells

#### Surface immobilized with AMPs

AMPs are short-cationic peptides known for their antibacterial activity against various Gram-positive and Gram-negative bacteria [88]. Unlike antibiotics, the antimicrobial activity of AMPs can be explained by the electrostatic binding between bacterial membrane and AMPs, which disrupts membrane integrity and leads to activation of autolytic enzymes; thus, they do not cause drug resistance in bacteria or activate adaptive immunity [146]. Moreover, the AMP coatings exhibit satisfactory biocompatibility, indicating that they have selective toxicity towards target microorganisms [147]. Various AMPs have been used as antibacterial coatings to prevent oral bacterial colonization and biofilm formation (Table 4) [46, 102, 148–150].

**Table 4:** Summary of the AMP coating

AMPs coating	Fabrication method	Bacteria affected	Antibacterial period	Cell response to the surface	In vivo study	References
GL 13 K	Covalent immobilization via silane-linker	*P. gingivalis*	5 d	Cytocompatible with HGF and MC3T3-E1	-	[148]
JH8194 and minTBP-1	Immobilization via Pro-rich linker PAPAP	*S. gordonii*, *S. sanguis*	6 d	No significant influence on the proliferation of MC3T3-E1	-	[149]
Hlf1-11	Immobilization via silanization or ATRP	*S. sanguinis, L. salivarius*, oral biofilm	4 weeks	Low cytotoxicity effect on HFFs	-	[150]
TBP-1-RGDS-hBD3-3	Anchoring via TBP-1	*S. gordonii*, *F. nucleatum*, *P. gingivalis*	72 h	No significant influence on the proliferation of MC3T3-E1	-	[46]
TBP-1-GGG-hBD3-3	Anchoring via TBP-1	*S. oralis*, *S. gordonii*, *S. sanguinis*	72 h	No significant cytotoxicity toward MC3T3-E1	-	[46]
Tet213	Layer-by-layer assembly technique	*S. aureus*, *P. gingivalis*	1 month	No significant affection on the viability of HaCaT	-	[102]

*AMPs* antimicrobial peptides, *PAPAP* pro-rich linker, *ATRP* atom transfer radical polymerization, *TBP-1* Engineered chimeric peptides containing Ti-binding fragments, *P. gingivalis Porphyromonas gingivalis, S. gordonii Streptococcus gordonii, S. sanguis Streptococcus sanguis, S. sanguinis Streptococcus sanguinis, L. salivarius Lactobacillus salivarius, S. oralis Streptococcus oralis, S. aureus* Staphylococcus aureus, *HGF* human gingival fibroblasts, *MC3T3-E1* mouse embryonic osteoblast precursor cells, *HFF* human gingival fibroblasts, *HaCaT* human immortalized keratinocytes

Generally, AMP coatings are prepared by physical anchoring or chemical covalent immobilization. Engineered chimeric peptides containing Ti-binding fragments that have a high affinity for the Ti substrates have been developed for physical anchoring [46, 151]. Though linkers or atom transfer radical polymerization, AMPs are covalently attached to the surface of Ti to achieve chemical immobilization [84, 148, 149]. Depositing the AMP-loaded coatings on Ti surfaces for subsequent release has proven to be an effective coating strategy. A layer-by-layer assembly technique was employed to form a multilayered coating as a delivery system for AMPs, providing antibacterial protection for up to 1 month [102]. Further investigations should focus on the in vivo antibacterial performance and osseointegration properties to realistically evaluate the efficiency of AMP-immobilized surfaces in the treatment of peri-implantitis.

### Surface modification with metal and/or metal oxide

The modification of dental implant surfaces with antibacterial metal elements and metal oxide nanoparticles represents an alternative strategy for preventing bacterial infection after implantation. Various ion implantation and coating techniques have been applied to modify Ti surfaces, which are listed in Table 5 [92, 93, 121, 152–165] and will be discussed separately in the following sections.

**Table 5:** Summary of the acting modes of different metal and/or metal oxide on antibacterial activity and osteogenic property

Material	Antibacterial mechanism	Bacteria affected	References
Ag	Directly interacts with bacterial cell membranes and causes interference in DNA transcription and cellular respiration. Released Ag ions can bind with thiol groups in proteins and inhibit respiratory enzymes, resulting in the production of ROS	*P. gingivalis*, *S. aureus*, *E. coli*, *A. actinomycetemcomitans*, *S. mutans*	[152–156]
Cu, CuO and Cu_2_O	The released Cu ions form a “safe zone” to improve implant healing. CuO_x_ can induce the generation of ROS to compromise membrane integrity and prevent biofilm development	*P. gingivalis*, *S. aureus*	[157–159]
Zn and ZnO	Induce the generation of ROS. Internalization into bacterial and disrupting bacterial membrane. Zn ion release disrupts enzyme system, amino acid metabolism	*S. aureus*, *E. coli*	[93, 160, 161]
CeO_2_	Electrostatic attractions between the positively charged CeO_2_ and the negatively charged bacterial cells. Interaction between CeO_2_ and thiol groups of bacterial cell surface proteins causes decreased membrane permeability	*E. faecalis*, *P. intermedia*, *P. gingivails*, *S sanguinis*, *F. nucleatum*	[92, 121, 162]
Ta	Formation of micro galvanic between the incorporated Ta and titanium consumes the transmembrane proton motive force, resulting in inhibited ATP synthesis. Nanostructured Ta surface could induce ROS generation to disrupt bacterial metabolism	*S. mutans*, *P. gingivalis*, *F. nucleatum*	[163–165]

*Ag* silver, *Cu* copper, *CuOx* cupric oxide or cuprous oxide, *Zn* zinc, *ZnO* zinc oxide, *CeO*_*2*_ cerium oxide, *Ta* tantalum, *DNA* deoxyribonucleic acid, *ROS* reactive oxygen species, *P. gingivalis Porphyromonas gingivalis, S. aureus Staphylococcus aureus, E. coli Escherichia coli, A. actinomycetemcomitans Aggregatibacter actinomycetemcomitans, S. mutans Streptococcus mutans, E. faecalis Enterococcus faecalis, P. intermedia Prevotella intermedia, S sanguinis Streptococcus sanguinis, F. nucleatum Fusobacterium nucleatum*

#### Silver

Silver (Ag) is recognized as the most effective antibacterial metal component and can provide a wide spectrum of antibacterial activity against various oral pathogenic bacteria [166]. Ag nanoparticles possess enhanced antibacterial properties, owing to their large total surface area and highly active surface for bacterial interaction, and have been widely applied for dental implant surface modification [167–169].

Numerous studies have demonstrated the ability of Ag nanoparticles coated surfaces to inhibit biofilm formation, and have optimized Ag concentration to achieve ideal cytocompatibility with osteoblast [170–172]. This consensus suggests that the antibacterial mechanisms of Ag nanoparticles are diverse, including contact sterilization and ion-mediated bactericidal effects. Ag nanoparticles can directly interact with bacterial cell membranes, and interfere with DNA transcription and cellular respiration so that to provide a satisfactory antibacterial effect [90, 173]. In addition, the released Ag ions can bind to thiol groups (-SH) of proteins, thereby inhibiting the synthesis of respiratory enzymes and leading to ROS generation [174, 175]. To further enhance the antibacterial period and biocompatibility, composite coatings of Ag nanoparticles were prepared on Ti-based surfaces by polydopamine [154] or layer-by-layer self-assembled chitosan-heparin [152]. These surfaces exhibit enhanced antibacterial properties without affecting cell viability. Since the biological function of Ag is dose-dependent, Ag nanoparticles in a suitable amount can promote osteogenic differentiation and improve bone fracture healing [176–178]. It is important to evaluate osteogenic properties via the optimization of Ag-containing concentrations.

Alternatively, bifunctional composite coatings containing bioactive materials and Ag were also developed. Polydopamine-induced nanocomposite coatings containing Ag and calcium phosphate (CaP) were applied onto the surfaces of TiO_2_ nanotubes using self-polymerized dopamine as a binder between the coatings and the substrate, the reducing agent and diffusion barrier for Ag nanoparticles, as well as an inducer of CaP biomineralization [156]. Because the uppermost CaP coating alleviated the adverse effects of Ag on the proliferation and viability of osteoblasts, the nanocomposite coating exhibited desirable antibacterial activity and in vitro cytocompatibility with MG63 cells. In addition, growth factors were co-loaded with Ag to enhance both antibacterial and osteogenic activities. Bifunctional coatings containing basic fibroblast growth factor (bFGF) and Ag were prepared on TiO_2_ nanotube surfaces using a polydopamine-heparin-assisted step-by-step cross-linking method [179]. The cross-linked coatings on the nanotextured surfaces promoted the slow release of bioactive bFGF. Along with Ag nanoparticles loading, the modified surface promoted osteogenic differentiation of dental pulp stem cells inhibited oral bacterial infections, and decreased the secretion of pro-inflammatory factors. Further investigations are required to evaluate the in vivo performance and long-term durability of these coatings.

#### Copper (Cu), cupric oxide (CuO) and cuprous oxide (Cu_2_O)

Cu, CuO, and Cu_2_O possess satisfactory antibacterial activities against a series of bacterial pathogens associated with peri-implantitis and have long been recognized as alternative antibacterial agents for Ag [180]. The attachment of CuO nanoparticles to bacterial cells can induce the generation of ROS, leading to an increase in intracellular oxidative stress [181]. In addition, the released Cu ions can be considered as potent antibacterial agents, because they can penetrate the bacterial cell membrane and disrupt enzyme functions, thereby achieving a bactericidal effect [182]. Furthermore, the appropriate amount of Cu ions can induce osteogenic differentiation and facilitate bone regeneration [183]. Therefore, Cu-derived materials are commonly used as an antibacterial candidate for the surface modification of dental implants. Cu nanocubes with an average size of 20 nm were deposited on the TiO_2_ substrates by pulsed electrodeposition [184]. The deposited copper oxide thin film exhibited high antimicrobial efficacy and drastically decreased bacterial adhesion. In another study, a spark-assisted anodization method in a composite deposition-anodization process was used to prepare Cu particle coating surfaces with varying Cu concentrations at the micro-nano scale [157]. The antibacterial efficiency was closely related to Cu concentration, and the surface with 7–9 µg Cu significantly reduced the viability of *P. gingivalis*. However, a high dosage of Cu can cause cytotoxic effects [185]. More biosafety tests including in vivo evaluations should be performed in order to ensure Cu-derived surfaces can protect against bacterial invasion in a biocompatible concentration range.

#### Zinc (zn) and zinc oxide (ZnO)

Zn and ZnO have a broad-spectrum of antibacterial functions and can be considered as candidates for the development of antibacterial surfaces [186]. The potent antibacterial actions can be summarized as follows. (1) The released Zn ions significantly affect the inhibition of active transport, and amino acid metabolism and damage the enzyme system. (2) ZnO nanoparticles can produce ROS, including hydrogen peroxide, hydroxyl radicals, and peroxide. (3) ZnO nanoparticles can disrupt cellular activities by precipitation onto the bacterial exterior or accumulating in the cytoplasmic area/periplasm space [187–189].

Zn-incorporated TiO_2_ surfaces can effectively inhibit the growth of both Gram-positive and Gram-negative bacteria, and the inhibition effect can be seen from the increase of Zn content [160]. The enhanced antibacterial properties and biological activity can be attributed to the sustained and slow release of Zn ions at low concentrations. Wang et al. [93] constructed a bilayer coating containing both ZnO nanorods and ZnO nanospheres (ZnO NRS). The nanorods were deposited onto the substrate via a hydrothermal method, and then the small-sized ZnO nanospheres were modified as the outermost layer. As a result, the small-sized nanospheres were rapidly released in the initial stage, while the nanorods were released slowly due to their larger particle size and stronger loading, thus achieving long-term antibacterial activity. In vivo, the antibacterial experiment was performed using a subcutaneous bacterial infection rat model. The ZnO NRS-modified samples exhibited the best antibacterial effects, which were manifested as the lowest number of bacteria detected by the plate colony counting method and the mildest degree of inflammation. After culture with human fibroblast cells up to 7 d, the surfaces showed mild cytotoxicity. The double-layered ZnO NRS structure has strong antibacterial activity and low cytotoxicity, so it can be considered a promising antimicrobial coating for peri-implantitis.

Since the biological effect of Zn ion release is dose-dependent, high concentrations of Zn ions can cause fatal toxicity and adverse effects on mammalian cells [190–193]. To prepare a cell-selective toxic surface with controllable Zn ion release, ZnO nanorod arrays were prepared on Ti surfaces by a hydrothermal method, and then ZnO was converted to ZnO@ZnS to form core-shell structured coatings [161]. The optimized curing treatment made the release of Zn ions more gentle, and the released concentration of Zn was significantly reduced from 3.5 mg/L to about 0.3 mg/L, but still had about 100% bactericidal effect. The ZnO@ZnS nanorod-array also optimized the release of Zn ion by depositing of highly-stable ZnS shell and promoted the attachment and migration of human gingival fibroblast cells. Further experiments should focus on the integration of ZnS coatings onto dental implants and to verify the biological response of different cell lines and the antibacterial effect against oral bacteria as well as osseointegration performance.

#### Cerium oxide

Cerium oxide (CeO_2_) has received increasing attention in the application of dental implants due to its osteogenic activity and antibacterial properties [92, 162]. Besides, CeO_2_ nanoparticles can be considered a promising candidate for scavenging ROS and reducing inflammation [194–196]. Due to the presence of oxygen vacancies in the crystal lattice, CeO_2_ nanoparticles enable cyclic redox reactions between Ce^3+^ and Ce^4+^ oxidation states, which help catalyze antioxidant properties and the ability to eliminate ROS.

**Fig. 6:**
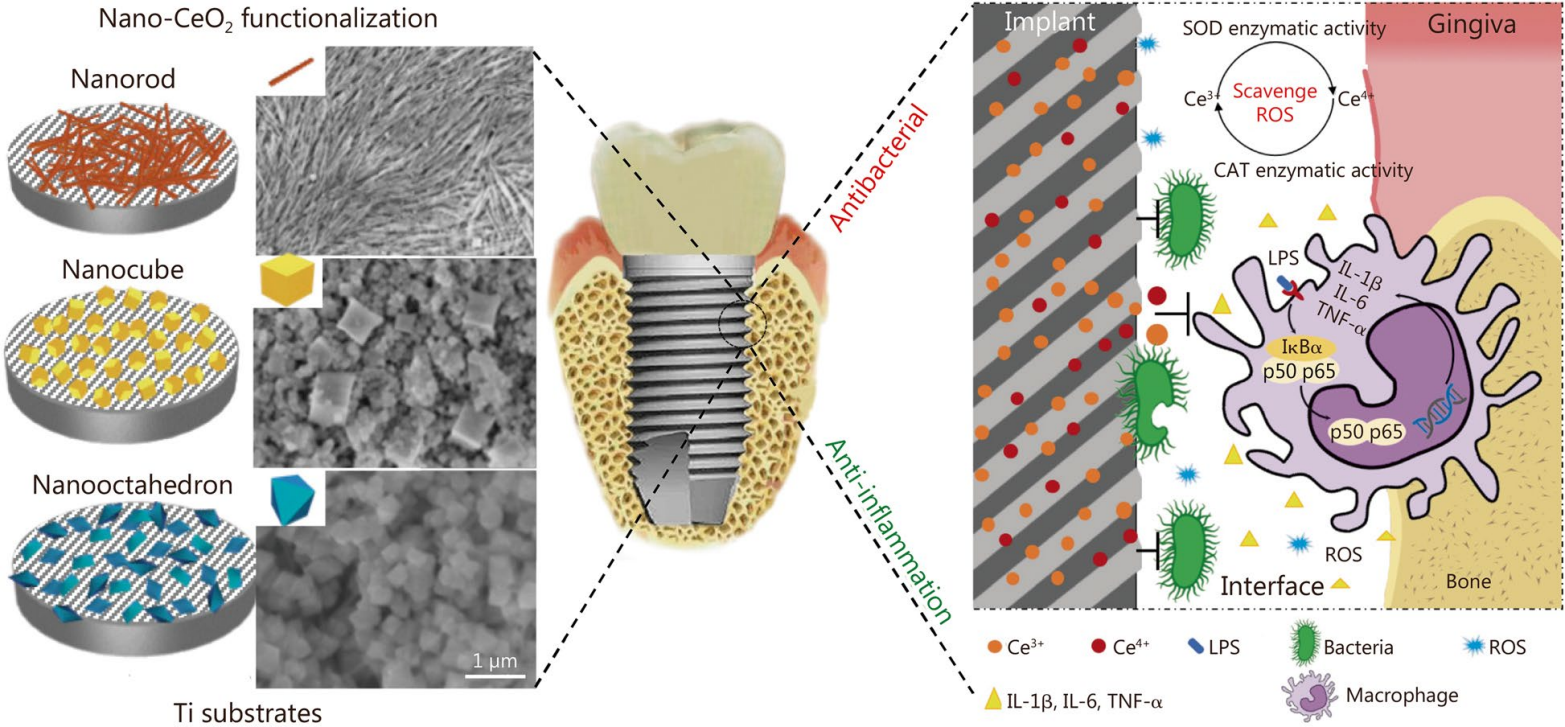
Schematic diagram of titanium substrate coated with different nanostructured CeO_2_ (nanorod, nanocube, and nanooctahedron) with the aim of enhancing the antibacterial and anti-inflammatory performance. The antibacterial effects can be attributed to the electrostatic interaction between nanostructured CeO_2_ and bacterial cell surface. And the anti-inflammatory effects can be attributed to the SOD and CAT mimetic activities [121]. Copyright 2019, Elsevier. Ce cerium, SOD superoxide dismutase, CAT catalase, LPS lipopolysaccharide, ROS reactive oxygen species, IL-1β interleukin-1β, IL-6 interleukin-6, TNF-α tumor necrosis factor-α

The amount and mobility of oxygen vacancies are closely related to the shape of CeO_2_ nanoparticles. Different shapes of CeO_2_, enclosed by specific crystal planes, affect the surface catalytic activity, resulting in different anti-inflammatory effects [197]. CeO_2_ with nanostructures of the rod (rod-CeO_2_), cube (cube-CeO_2_), or octahedron (octa-CeO_2_) were prepared using a hydrothermal method under different synthetic conditions [121]. All three nanostructured CeO_2_ exhibited ROS-scavenge activity, which is characterized by superoxide dismutase (SOD)- and catalase (CAT)-mimicking activities (Fig. 6). As the most planes of the three groups, octa-CeO_2_ exhibited the highest Ce^3+^ levels and superior ROS scavenging performance. To further evaluate the antibacterial and anti-inflammatory properties, the three nanostructured CeO_2_ were coated onto Ti surfaces via a spin coating method. In vitro and in vivo studies revealed that all three surfaces exhibited strong antibacterial activity, characterized by inhibition of early bacterial adhesion and biofilm formation. Unlike the above metal and metal oxide nanoparticles, the antibacterial mechanism of CeO_2_ might be ascribed to the electrostatic interactions between CeO_2_ and bacteria as well as the binding between CeO_2_ and the thiol groups of bacterial cell surface proteins [198]. Besides, the modified surface of octa-CeO_2_ showed the strongest anti-inflammatory properties among all tested groups due to the smallest particle size and octahedral structure exposing more crystalline planes, thereby significantly reducing the mRNA expressions of IL-1β, IL-6, and TNF-αin the tissue around the implanted Ti disks.

#### Tantalum

Tantalum (Ta) is another promising metallic component for dental and orthopedic applications because of its excellent osteogenic and antibacterial properties [24, 163, 165, 199–202]. Zhu et al. [164] synthesized hierarchical micro-nanostructured surfaces by depositing Ta films onto acid-etched Ti using magnetron sputtering to achieve the selective bactericidal effect. The Ta film with nanostructured (20–50 nm) effectively inhibited the adhesion and growth of *S. mutans* and *P. gingivalis*.

The underlying molecular mechanisms of antibacterial activity can be attributed to the formation of micro-galvanic between the incorporated Ta and Ti substrates, leading to the inhibition of ATP synthesis. Additionally, the nanostructured Ta surface induced ROS generation, thereby promoting lipid peroxidation of the cell membrane and reducing catalase activity and glutathione levels, resulting in the disruption of bacterial metabolism. Importantly, a in vivo biological study has shown that the Ta-coated Ti surfaces significantly promote bone-implant integration by stimulating the expression of bone-forming proteins [165].

### Nitride ceramics coatings

Considering the intrinsic mechanical properties, desired biocompatibility, and biological activity, nitride ceramics can be regarded as novel biomaterials for dental implant applications [203, 204]. Examples include silicon nitride as the implant or prosthetic crown [205, 206]; chromium nitride and niobium nitrides abutment coating [204]; TiN and tantalum nitride coatings for dental implant [202, 207, 208]. Among these, TiN, which has been widely studied for decades, is the most commonly used material for making protective coatings for dental implants.

Corrosion of Ti-based dental implants may adversely affect the peri-implant tissue and increase the risk of biofilm accumulation [209, 210]. TiN is characterized by good biocompatibility, high hardness, and chemical stability, so it can be applied as a protective coating for Ti and Ti-6Al-4 V alloys to enhance their tribology performance and corrosion resistance [211–215]. Generally, TiN coatings are prepared through physical vapor deposition or surface nitriding. The deposited TiN coating showed good anti-adhesion against various oral bacteria in vitro and in vivo [82, 83, 216–219]. To further enhance the antibacterial activity, the TiN coatings were modified via a quaternization reaction. The quaternized TiN surface effectively inhibits bacterial adhesion and biofilm formation, which may be attributed to the disruption of the bacterial cell wall by quaternary nitrogen atoms, leading to the leakage of cell substances and ultimately apoptosis of bacteria [218, 220].

Metal ion co-implantation is another promising modification strategy for improving the antibacterial performance and biological activity of TiN coatings. Metal ions, including Zn/Ag [221], Ag/Ca [222], Mg/Ag [223], or Cu/Zn [224], are co-implanted onto TiN-coated Ti-6Al-4 V surfaces via a plasma immersion ion implantation and deposition (PIII&D) system. An ideal balance between antibacterial activity and osteogenic ability can be achieved by adjusting the metal ion ratios and deposition parameters. Based on the above features and advantages, TiN can be considered a promising candidate for dental implant coating material for the prevention of peri-implantitis. Further investigations should focus on in vivo studies and in-depth research of antibacterial and osteogenic mechanisms.

### Surface modification with G-based materials

G is a typical nano-sheet material with an atomic thickness, composed of sp^2^-hybridized carbon atoms arranged in a two-dimensional honeycomb lattice structure. Owing to their satisfactory antibacterial performance and osteogenic activity, G-based nanomaterials have been widely used for implant surface coatings. Their modification strategies are summarized in Table 6 [94–96, 225–233].

**Table 6:** Summary of G-based coatings and the corresponding biological effects

Material type	Modification method	Bacterial affected	Cell response to the surface	In vivo study	References
Single-layered G	Airbrush spraying	*S. aureus*	-	-	[225]
Single-layered G	CVD-grown and vacuum-assisted dry transfer technique	*S. mutans*, *E. faecalis*, *C. albicans* and *P. aeruginosa*	-	-	[226, 227]
Single-layered G	PMMA-assisted transfer and thermal treatment	*E. coli* and *S. aureus*	Enhanced cell adhesion and osteogenic differentiation	Increased ectopic bone formation in nude mice model	[95]
Single-layered G	PMMA-assisted coating	-	-	Enhanced osseointegration in rabbit femurs	[228]
GO	Atmospheric plasma deposition	*S. mutans* and *P. gingivalis*	Enhanced cell proliferation and osteogenic differentiation	-	[229]
GO/Carbon fibers/PEEK	Electrostatic powder spraying	*S. aureus*	Good cytocompatibility	-	[94]
GO/Chitosan/Hydroxyapatite	Electrophoretic deposition	-	Enhanced proliferation and differentiation of BMSCs	Enhanced osseointegration in rat model of tibia bone defect	[96]
GO/Minocycline hydrochloride	APTES-assisted coating method	*S. aureus*, *E. coli* and *S. mutans*	Good cytocompatibility	-	[232]
GO/Ag nanoparticles	Electroplating and UV reduction methods	*S. mutans* and *P. gingivalis*	Mild cytotoxicity	-	[233]
rGO	APTES-assisted coating method	-	Accelerated BMSC osteogenic differentiation	-	[230]
rGO/Dexamethasone	APTES-assisted coating method	-	Enhanced differentiation of MC3T3-E1	-	[231]

*G* graphene, *GO* graphene oxide, *rGO* reduced graphene oxide, *CVD* chemical vapor deposition, *PMMA* polymethyl methacrylate, *APTES* (3-aminopropyl)triethoxysilane, *UV* ultraviolet, *S. aureus Staphylococcus aureus, S. mutans Streptococcus mutans, E. faecalis Enterococcus faecalis, C. albicans Candida albicans, P. aeruginosa Pseudomonas aeruginosa, E. coli Escherichia coli, P. gingivalis Porphyromonas gingivalis, BMSCs* bone marrow mesenchymal stem cells, *MC3T3-E1* mouse embryonic osteoblast precursor cells

Generally, G-based nanomaterials used for dental implant surface modification can be divided into three categories — single-layered G, graphene oxide (GO), and reduced GO (rGO). The antibacterial mechanism of G materials can be attributed to the physical disruption of the bacterial membrane [94, 95, 225] and the reduction of surface free energy [226]. In addition to the “nano-knife” effect of penetrating bacterial cell membranes, oxidative stress induced by ROS generation plays an important role in the antibacterial performance of GO materials [234–236]. G-based coatings also exhibit enhanced osteogenic properties in vitro [94, 229, 230] and in vivo [95, 96, 228]. A mediator (polymethyl methacrylate)-assisted transfer technique was used to transfer a single-layer of G onto a smooth titanium plate, which was then thermal treatment to improve the adhesion between G and Ti substrate [95]. The incorporation of G onto the Ti substrate effectively promoted the adhesion of human adipose-derived stem cells and human bone marrow-derived mesenchymal stem cells (MSCs). Additionally, G-based nano-sheets are promising carriers for loading of osteogenic dexamethasone [231], minocycline hydrochloride [232], or Ag nanoparticles [233] to endow the surface with antibacterial activity and promote bone-implant integration. However, the biosafety issue of G-based nano-materials cannot be ignored due to the cytotoxic and antibacterial effects of GO [237]. Moreover, the binding stability between G-based coatings and Ti substrates should be evaluated in clinical operation, and long-term safety should be further tested in a complex oral environment.

### Surface modification with polymer materials

#### Synthetic polymers

##### Polymer coatings with anti-adhesion properties

Bacterial adhesion to the surface of the implant is the first stage of oral biofilm formation, leading to sustained tissue inflammation around the implants and ultimately implant failure [70]. Thus, developing functional surfaces that resist bacterial adhesion and prevent biofilm formation is crucial for preventing peri-implantitis. The use of anti-fouling polymers as surface coatings is an effective antibacterial strategy for dental implants, as these polymers prevent protein adsorption and subsequent bacterial adhesion.

Poly(ethylene glycol) (PEG) is a well-known hydrophilic polymer that can effectively reduce protein adhesion and bacterial attachment owing to its highly hydrated and flexible molecular chains [238–240]. Harris et al. [80, 241] modified the TiO_2_ surface with poly(L-lysine)-grafted-PEG copolymer coating. In this approach, poly(L-lysine) was first bound to the substrate and served as the backbone for subsequent PEG side chain immobilization. The copolymer coating effectively reduced the non-specific adsorption of blood components and extracellular matrix proteins, thereby reducing bacteria adhesion. However, PEG modification also affected cell attachment and adhesion, which could be restored by introducing Arg-Gly-Asp bioactive peptides on the anti-adhesive copolymer coating. Buxadera-Palomero et al. [84] constructed a PEG-like anti-fouling coating via plasma polymerization. The coating exhibited satisfactory bacterial resistance with low adhesion to *Streptococcus sanguinis* and *Ligilactobacillus salivariu*. Since changing the parameters in plasma polymerization resulted in different surface properties, and the application of higher powers can lead to surfaces with good cell-adhesive properties, the plasma polymerized PEG-like coating showed adequate cell response. The adhesion and morphology of osteoblasts and fibroblasts on the coated samples were similar to those on the Ti surfaces.

##### Modification with polymer materials

 Coating with amphoteric hydrophilic polymers is another surface modification strategy to prevent biofilm formation. The antibacterial mechanism of amphoteric polymer is mainly attributed to the bactericidal effect of direct contact caused by the destruction of the cell membrane. Kaleli-Can et al. [242] used sed plasma polymerization to construct an amphoteric diethyl phosphite (DEP) coating on the Ti surface. Due to the formation of acidic and basic groups after plasma deposition, the DEP coating exhibited enhanced surface hydrophilicity, roughness, and pH-related interfacial attractiveness. Bacterial and fungal assays demonstrated that the adhesion of *S. aureus* to *Candida albicans* was not completely inhibited in the initial stage. However, a significant decrease in the abundance of live pathogens rather than multiplication was observed with the prolonged incubation time, indicating satisfactory contact sterilization ability provided by amphoteric DEP coating.

##### Polymer coatings as drug-delivery systems

To further improve the bactericidal effect, synthetic polymer coatings have been applied to drug-delivery systems. For example, poly(L-lactic acid) nanoparticle coatings [98, 243] and layer-by-layer deposited poly(acrylic acid)-poly-L-lysine coatings [244], have been used to load and release numerous antibacterial agents, or directly as the antibacterial agents [137] to prevent peri-implant diseases. The explosive release of antibiotics in the initial stage may result in poor antibacterial effect in preventing bacterial invasion, leading to implantation failure. Therefore, a long-acting renewable polymeric coating loaded with chloramine (Ti-PAA-NCl) was prepared to prevent and treat peri-implantitis [105].

**Fig. 7:**
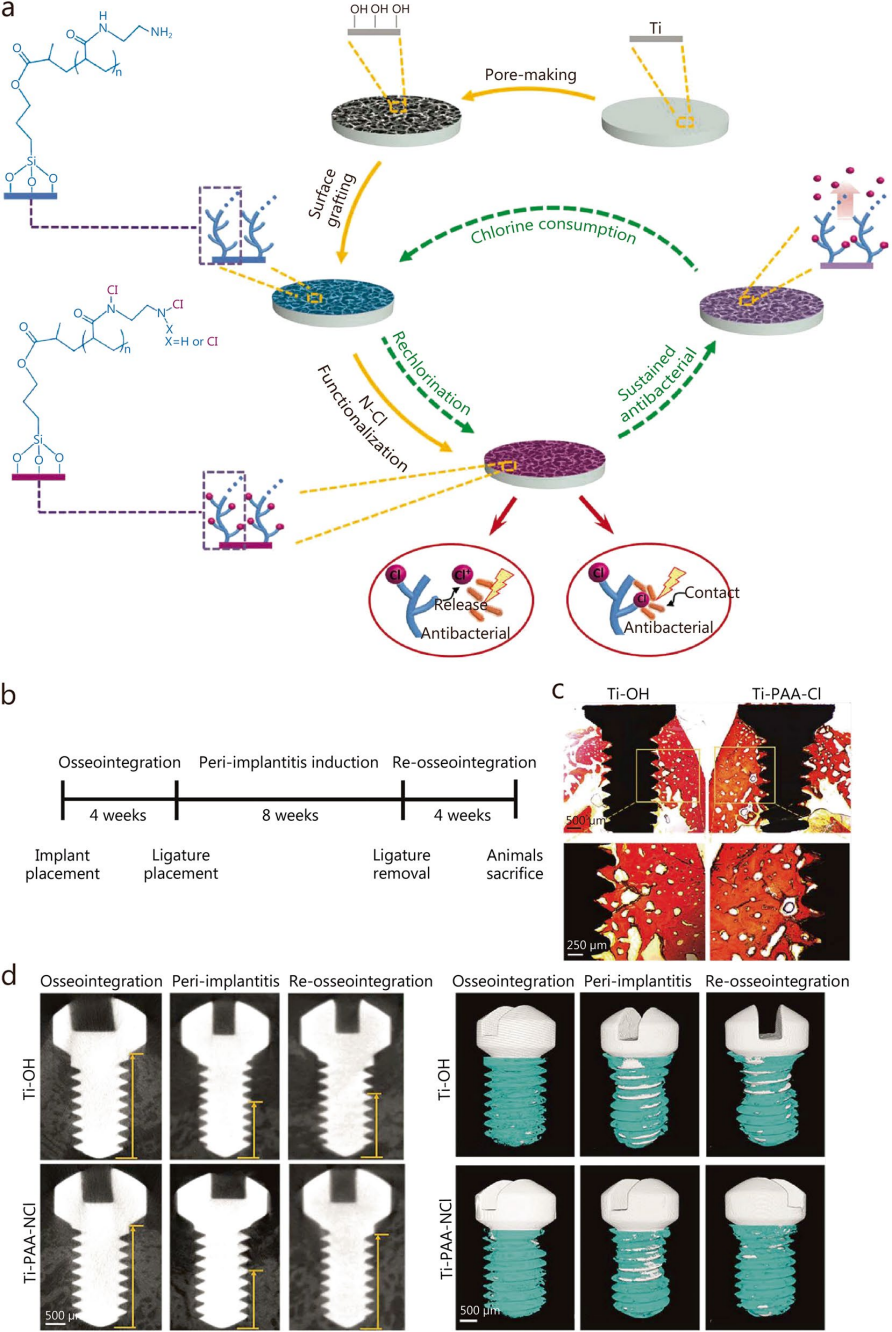
The preparation and in vivo assessments of osseointegration and anti-infection ability of Ti-PAA-NCl. **a** Schematic diagram of the synthesis of Ti-PAA-NCl coating on the Ti substrates. **b** Time period of experiments. **c** New bone formation via Van Gieson’s staining after 4 weeks of implantation (scale bar is 500 μm in the top image and 250 μm in the bottom one). **d** Micro-CT images of the bone height surrounding the implants after osseointegration for 4 weeks, peri-implantitis for 8 weeks, and re-osseointegration for 4 weeks (scale bars is 500 μm). **e** Micro-CT three-dimensional reconstructions of the implants and surrounding bone tissues (scale bars is 500 μm) [105]. Copyright 2021, the author(s). Ti titanium, N-Cl nitrogen-halamine, Ti-OH alkali-heated titanium disks, Ti-PAA-Cl polymeric coating loaded with chloramine

As shown in Fig. 7a, the antibacterial coating was prepared through surface pore formation, poly(acrylic acid) grafting, and N-chloramine functionalization. The obtained coating exhibited high antibacterial activity against single key pathogenic bacteria (*S. aureus*, *P. gingivalis*) and composite oral colonies from patients suffering from peri-implantitis. The key component for the bactericidal function was the active halogen (i.e. Cl^+^) in N-chloramine, which eradicated bacterial infections through the synergistic effect of contact sterilization and release-mediated bactericidal action. After consumption, the polymeric coating could be effectively regenerated by simple rechlorination. The antibacterial performance was also investigated in vivo using a rabbit model of peri-implantitis and the oral environment of patients [105]. Results demonstrated that the Ti-PAA-NCl coatings effectively reduced biofilm mass and provided prolonged protection for bone regeneration during healing. According to Van Gieson’s staining, the mini-implants coated with Ti-PAA-NCl formed satisfactory osseointegration after 4 weeks of implantation (Fig. 7b-c). Furthermore, two-dimensional analysis and three-dimensional reconstruction of micro-CT data showed that Ti-PAA-NCl successfully promotes the recovery of bone tissue that was previously absorbed in peri-implantitis. (Fig. 7d-e).

Enhanced osseointegration and sustained antibacterial activity are important for successful implantation. Therefore, the application of dimethylaminododecyl methacrylate (DMADDM)-loaded poly(amidoamine) (PAMAM) coating on the surface of macro-arc oxidation (MAO) treated Ti implant surface can effectively prevent infection while promoting osseointegration [101]. The PAMAM dendrimer exhibited desirable biocompatibility and facilitated cell adhesion and the formation of hard tissue. The dendrimer PAMAM cavity also exhibited a desired drug loading capacity, making it suitable as a carrier for antibacterial agents to alleviate DMADDM-associated cytotoxicity. In highly infected environments, the polymeric coating effectively inhibits biofilm formation by controlled release of DMADDM after implant surgery both in vitro and in vivo. Additionally, the PAMAM-DMADDM coating combined with MAO treatment showed excellent anti-infective and osteoconductive characteristics in a rat model of peri-implantitis in vivo.

#### Surface modification with naturally derived polymers

Chitosan, a naturally derived linear cationic polysaccharide, consists of glucosamine and N-acetylglucosamine. Chitosan has been widely applied in dental implantology owing to its superior biocompatibility, biodegradability, bioactivity, and antibacterial activity [245]. Modification of Chitosan can enhance antibacterial efficacy against Gram-positive and Gram-negative bacteria on implant surfaces [246, 247]. Govindharajulu et al. [103] used a layer-by-layer assembly technique to construct a chitosan-based coating on the Ti surface, which showed significant antibacterial activity against *S. gordonii*. The antibacterial effect of chitosan is attributed to the electrostatic interaction between chitosan and bacterial cell membrane, leading to permeabilization of the bacterial cell surface and subsequent leakage of intracellular components, ultimately resulting in bacterial death [248]. Additionally, the abundant amino groups present in chitosan are believed to exert bactericidal action [249]. *S. gordonii* is known to be a primary colonizer of oral tissues and provides a growth substrate in the oral cavity that facilitates adhesion for biofilm formation by *P. gingivalis* [250]. Therefore, the inhibition of *S. gordonii* growth can effectively prevent colonization by *P. gingivalis* [251]. Moreover, chitosan-coated dental implants demonstrate the desired capacity to promote new bone formation, making chitosan a promising candidate for developing implants with both antibacterial and osteogenic properties [252].

Polysaccharides have also been used as fundamental components for the development of drug delivery systems or loaded with antibacterial metal nanoparticles to inhibit biofilm formation on dental implants [152, 253, 254]. Chitosan and alginate are recognized as desired polycations and polyanions capable of forming polyelectrolyte complex multilayers by consecutive adsorption through electrostatic interactions. Lv et al. [136] employed a layer-by-layer self-assembly strategy to develop multilayered chitosan and alginate coatings with minocycline on Ti. Glutaraldehyde was used for covalently immobilizing chitosan onto the surface of amino-functionalized Ti substrate as the primary layer, ensuring the overall stability of the coating. Subsequently, a cycled multilayer construction was performed. The incorporation of multilayer coatings significantly increased the loading capacity of minocycline, resulting in sustained release over a period of 14 days to effectively inhibit the pathogenic bacteria adhesion. Additionally, the antibacterial performance of these coatings was also attributed to their surface charge and hydrophilicity, as well as the inherent antibacterial ability possessed by chitosan itself, which remained effective even after cessation of minocycline release.

### Photosensitive material coating

In recent years, phototherapy has drawn increasing attention due to its potential to effectively eradicate bacteria without inducing drug resistance, thereby advancing conventional antibiotic methods. Pathogenic microorganisms can be eradicated through the generation of ROS or heat via photoirradiation, depending on the photosensitive materials used in phototherapy [255]. TiO_2_ is typically classified as an N-type semiconductor owing to its oxygen deficiency, photocatalytic properties, and photoactivity [256]. Under ultraviolet light irradiation, anatase TiO_2_ coating can release ROS (•OH, O_2_^–^, HO_2_^–^, and H_2_O_2_) to eliminate attached bacteria [257–259]. However, ultraviolet light exerts detrimental effects on living organisms and has a very limited tissue-penetration depth. Several surface modification strategies, such as doping with noble metal (Ag or Au) nanoparticles or nitrogen (N), have been employed to enhance the photocatalytic activity of TiO_2_. Consequently, under visible light irradiation, the generated ROS can effectively eradicate the attached oral bacteria and inhibit biofilm formation, thus improving the success rates of dental implants [260–263]. Nevertheless, the restricted tissue penetration capability of visible light may impede its further application as a therapeutic agent for photodynamic therapy (PDT) [264].

**Fig. 8:**
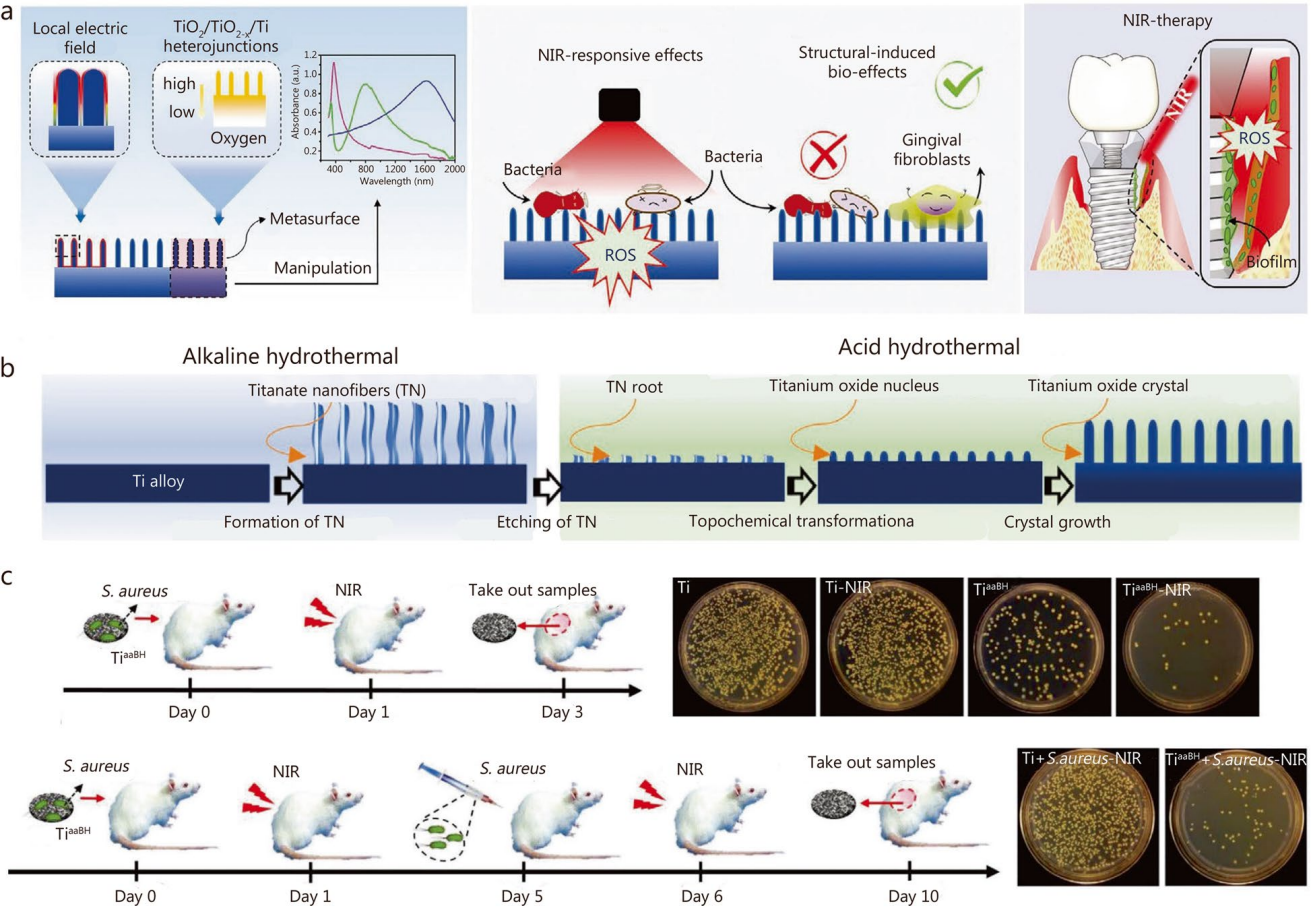
The preparation and in vivo anti-bacterial performance of quasi-periodic titanium oxide metasurface. **a** Schematic diagram of the design principle for the aaBH method to use metasurfaces to endow the implant with potent NIR-responsive antibacterial activity. **b** The aaBH method to construct quasi-periodic titanium oxide metasurface on Ti alloy implants. **c** The in vivo animal model with one and two injections of bacteria. Copyright 2021 [111]. TiO_2_ titanium dioxide, Ti titanium, TN titanium dioxide nanorods, NIR near-infrared, *S. aureus Staphylococcus aureus*, ROS reactive oxygen species

The NIR light has a strong tissue-penetrating effect, with relatively low adsorption of water and interstitial fluid in organisms. It has been widely utilized in PDT and photothermal therapy [113, 265–267]. However, the photoactivation of TiO_2_ requires UV light because of its wide bandgap. To reduce the bandgap and increase the NIR adsorption of pristine TiO_2_, a quasi-periodic metasurface was constructed on a Ti alloy implant using an alkaline-acid bidirectional hydrothermal (aaBH) method (Fig. 8a) [111]. The Ti surface underwent hydrothermal treatment to form sodium titanate nanofibers, followed by another round of hydrothermal treatment to provide Ti ions for the growth of TiO_2_ crystals (Fig. 8b). Under the acidic condition, the titanate root underwent the topochemical transformation from titanate to titanium oxide crystallites, which then acted as seeds and growth to rod-shaped crystals by consuming the Ti ions in the solution. The reaction time and acid concentration were critical factors that affected the dimension and size of the nano-structural unit in the metasurface, which in turn manipulated the light adsorption. The metasurface subjected to a 4-hour acid treatment exhibited remarkable selectivity in adsorbing NIR and potent photocatalytic activity upon NIR light irradiation, as evidenced by the generation of oxygen (^1^O_2_) and hydroxyl radical (·OH). Both in vitro and in vivo investigations confirmed its superior antibacterial efficacy under NIR irradiation (Fig. 8c). Additionally, the designed nanostructure exhibited promising biological enhancement effects, as indicated by the upregulation of adhesion-related gene expression in human gingival fibroblasts, thus proving its potent nanostructure-induced biological effects. Furthermore, studies have revealed that the formation of a TiO_2_ metasurface imparts NIR-responsive antibacterial functions to Ti alloys and induces a certain degree of antibacterial activity, highlighting the multi-functionality of this metasurface.

### Antibacterial alloys

Commercially available dental implant materials, including pure Ti and Ti-6Al-4 V, are susceptible to bacterial infections because they lack antibacterial properties [268]. Many studies have focused on alloying antibacterial metal elements with Ti to enhance the antibacterial performance [269, 270]. Ti-Cu alloys have emerged as prospective dental materials for the prevention of bacterial infections [108]. The antibacterial properties of Ti-Cu alloys vary depending on the Cu concentration, with higher Cu content exhibiting better antibacterial activities than those with lower Cu content [34, 271, 272]. The Ti-Cu alloys were treated with sandblasting and acid etching (SLA) to combine chemical design and micro-submicron hybrid structures, to enhance osteogenic activities while preserving bacterial inhibitory performance [106]. The SLA-treated Ti-Cu alloys (Ti-Cu/SLA) exhibited significant promotion of inhibitory effects against oral anaerobic bacteria and enhanced expression of osteogenesis-associated genes. Furthermore, the Ti-Cu/SLA implant demonstrated the ability to prevent bone resorption caused by bacterial infection and promote osseointegration. Numerous studies have consistently demonstrated the superior capacity of Ti-Cu alloys in preventing bone resorption induced by bacterial infection. Liu et al. [273] conducted a systematic investigation of the anti-infection ability and biocompatibility of Ti-Cu alloys using ligature- and sucrose-rich diet-induced models. Compared to pure Ti implants, Ti-Cu alloys exhibited the potential in preventing infections and excellent biocompatibility. The anti-infection mechanism of Ti-Cu alloys is believed to involve the maintenance of homeostasis in oral microbiota. Carbohydrates present in dental plaque formed on the implant surface were metabolized through tricarboxylic acid cycles, effectively inhibiting the formation of an acidic environment and reducing accumulation of acidogens and pathogens, thereby maintaining a balance microflora between aerobic and anaerobic bacteria.

## Strategies to enhance zirconia dental implant success

**Fig. 9:**
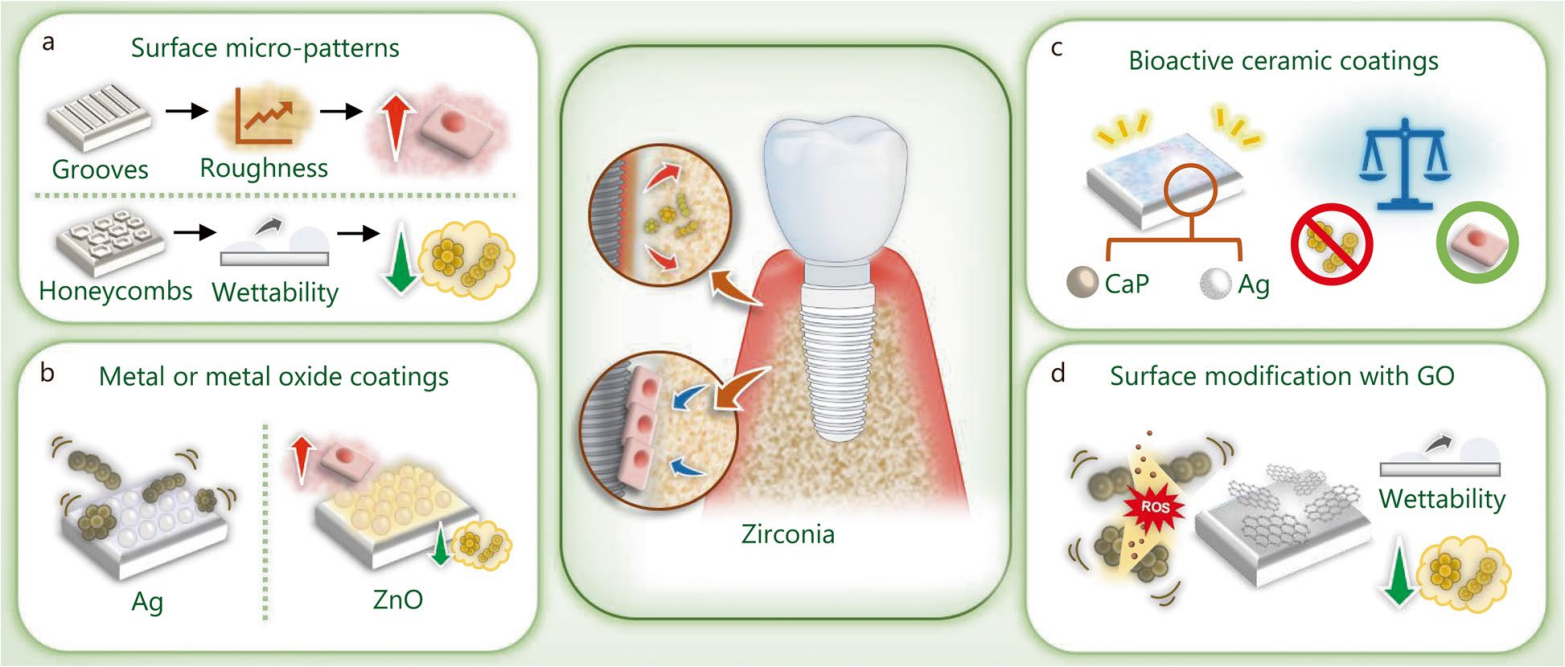
Biomaterials and modification strategies for zirconia dental implant to enhance the antibacterial properties and osteogenic activities. **a** Modification of the surface with micro-patterns. **b** Coating the surface with metal or metal oxides. **c** Modification of the surface with bioactive ceramic coatings. **d** Coating the surface with GO. Ag silver, ZnO zinc oxide, GO graphene oxide, CaP calcium phosphate

Over the last decade, metal-free bioceramics, particularly yttria-stabilized tetragonal zirconia, have emerged as alternative candidates for dental implants. The use of zirconia in dental implant material can help prevent metal corrosion-related implant failures and the immune-mediated surrounding tissue reactions caused by the accumulation of metallic particles. Zirconia has several advantages such as low modulus of elasticity, superior biocompatibility, and mechanical stability [24]. Additionally, the ivory color of zirconia also meets the increasingly esthetic requirements of dental implants. Zirconia dental implants, such as WhiteSky (Bredent GmbH & Co. KG, Senden, Germany) and the Zit-Z systems (Ziterion GmbH, Uffenheim, Germany), have been marketed for clinical use [274]. Surface roughness is improved by sandblasting combined with acid etching to achieve better osseointegration [275]. Zirconia dental implants have shown satisfactory clinical success, with a lower affinity for dental plaque accumulation than Ti-based dental implants [276, 277]. However, the application of zirconia dental implants is limited due to surface modification challenges [278]. Therefore, various surface engineering strategies (Fig. 9a) and coating techniques (Fig. 9b-d) have been applied to endow zirconia with enhanced antibacterial properties to prevent peri-implant inflammation.

### Surface micro-patterns

Grit blasting and acid etching are commonly used strategies to modify the morphology and bioactivity of zirconia dental implants. However, the aluminum and fluorine residues generated during the modification procedure may result in contamination and poor in vivo performance [279]. Therefore, the femtosecond laser etching technology was introduced to prepare microgroove patterns with nanoscale spherical structures on the surface of zirconia [280]. This microgroove structure promotes cell adhesion, proliferation, and differentiation in vitro and reduces stress on cortical bone and osteophyte in vivo. To enhance the antibacterial properties, laser surface texturing was used to prepare micro-honeycombs on the zirconia specimens. Wettability plays a crucial role in the antibacterial behavior of textured zirconia ceramics, and hydrophobic surfaces are more conducive to inhibiting bacteria adhesion, extension, and reproduction, thereby achieving excellent antibacterial performance [71]. The introduction of a micro-honeycomb texture effectively improves the surface hydrophobicity, thereby enhancing the antibacterial performance [281]. Although the stronger the hydrophobic surface, the stronger the antibacterial properties, because the surface texture affects the smoothness of the material, it is also conducive to the accumulation and reproduction of bacteria in the concave and corner areas.

### Metal or metal oxide coatings

Zirconia is widely used in the manufacture of dental implants due to its esthetic and biocompatibility, but zirconia is commonly recognized as a nearly inert biomaterial. Therefore, metal or metal oxide nanoparticles are introduced to enhance the antibacterial properties through contact sterilization and ion-mediated bactericidal effects.

Ag nanoparticles were incorporated onto the surface of yttria-stabilized zirconia at different nanoparticle concentrations. Ag nanoparticle-coated surfaces exhibited broad-spectrum antimicrobial activity against oral bacteria (*S. mutans* and *Aggregatibacter. actinomycetemcomitans*) and bacteria associated with orthopedic implant-related infections [282]. However, Ag nanoparticle-coated surfaces exhibited dose-dependent cytotoxicity, with lower Ag concentrations showing higher cell viability. To better balance the antibacterial activity and cytocompatibility, the concentration of Ag nanoparticles was optimized in the range of 0.2–2.5 mmol/L, which corresponds to Ag weight densities of 2.6–32.0 µg/cm^2^. Further evaluation should focus on the lone-term effects of Ag nanoparticle coatings on osteoblast proliferation, differentiation, and in vivo osseointegration.

**Fig. 10:**
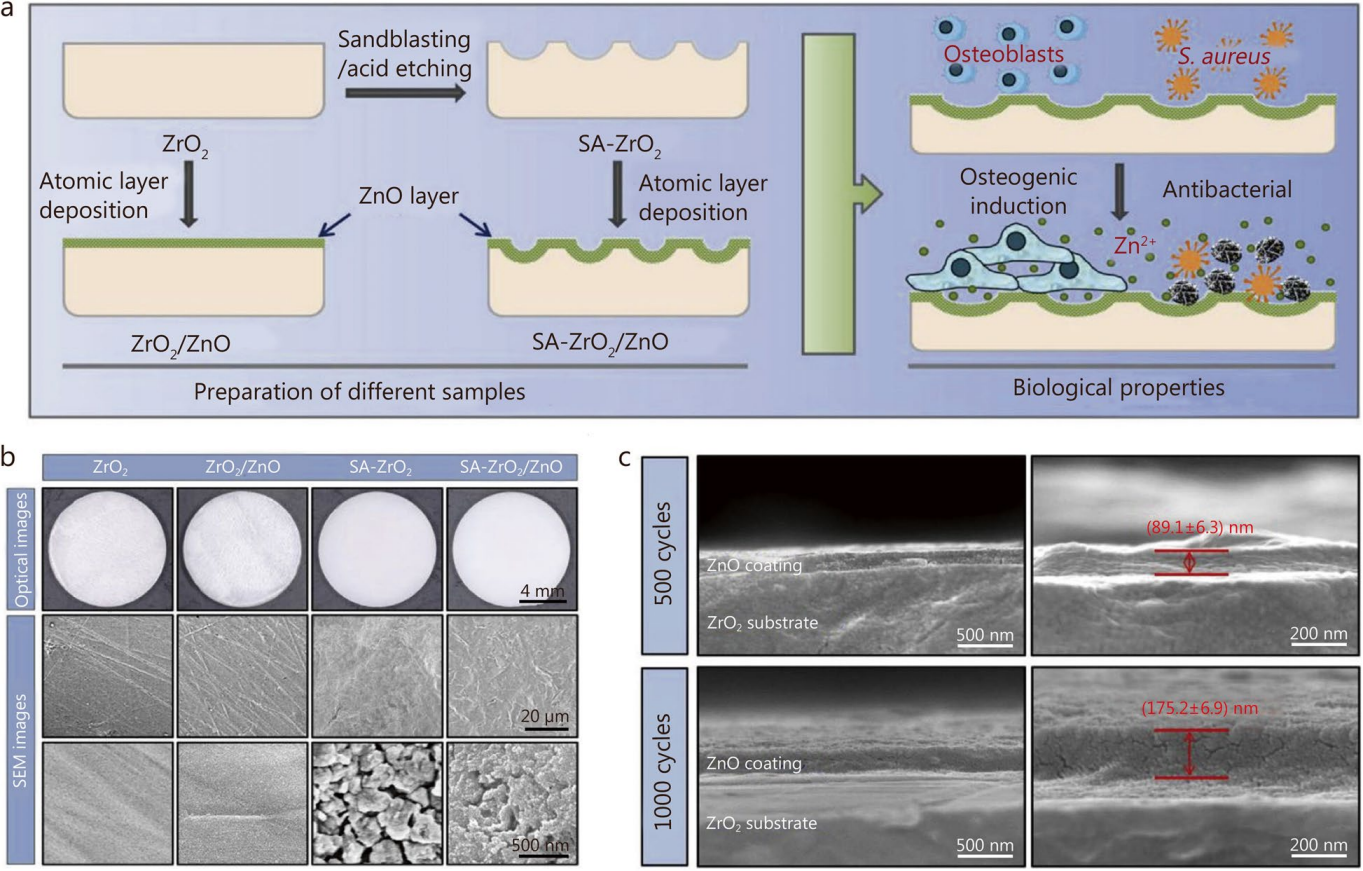
ALD of ZnO on microrough zirconia. **a** Schematic diagram of the preparation and biological properties of different samples. **b** The optical and SEM images of four samples. **c** Cross-sectional SEM images of ZnO layers prepared by 500 and 1000 ALD cycles [287]. Copyright 2019, Elsevier. ALD atomic layer deposition, ZnO zinc oxide, SEM scanning electron microscopy, ZrO_2_ zirconium dioxide, SA and blasted and acid etching, *S. aureus Staphylococcus aureus*

ZnO has broad-spectrum antibacterial efficacy and can be used on the surface of zirconia dental implants to prevent bacterial infection [186, 283]. The biological function of Zn ion release is dose-dependent, with low doses of Zn ion effectively enhancing osteogenic induction, while high doses (more than 0.02 mg/L) lead to osteocytotoxicity [284–286]. To simultaneously enhance the osteogenic properties and antibacterial activity of zirconia surfaces, a method combining grid blasting, acid etching, and atomic layer deposition (ALD) was used to prepare ZnO-coated microrough structures on zirconia substrates. With the increase of ALD cycles, the survival rate of MC3T3-E1 cells increased, among which the zirconia surface after 30 ALD cycles exhibited the highest cell viability, which was selected as the target material for further investigations. As shown in Fig. 10, after ALD treatment, a nano-grained ZnO layer was uniformly deposited on the micro-pits of zirconia surfaces. The antibacterial activity of ZnO coatings against *P. gingivalis*, *S. aureus*, and *E. coli* lasted for more than 14 d [287]. In addition, the ZnO-coated zirconia surfaces were also conducive to the proliferation and osteogenic differentiation of MC3T3-E1 cells owing to the synergistic effects of the micro-nano structured morphology and the release of Zn ions.

### Bioactive ceramic coatings

Bioactive CaP coatings have been introduced on zirconia surfaces to enhance interfacial binding [288]. Goldschmidt et al. [289] used a biomimetic precipitation technique to produce CaP and Ag nanoparticle coatings onto zirconia surfaces, to endow the surface with both bioactivity and antibacterial properties. However, higher concentrations of Ag resulted in cytotoxicity; only samples containing 0.05% (atom) Ag nanoparticles showed cytocompatibility. As the size and concentration of Ag nanoparticles are closely related to the cytotoxic effect, further research should focus on the “therapeutic window” between antibacterial activity and cytocompatibility [290].

### Surface modification with GO

Compared with pristine G nano-sheets, GO has a hydrophilic tendency because of its surface-rich carboxyl, hydroxyl, and epoxy functional groups. Numerous studies have demonstrated the superior abilities of GO-modified surfaces to resist bacterial infections and promote bone regeneration through osteoblast activation [291–293]. Therefore, GO was deposited onto zirconia surfaces to determine its effect on bacterial adhesion and osteoblast activation. Zirconia substrates were coated with GO using an atmospheric-pressure plasma generator, resulting in the formation of coatings that exhibited a cloudy appearance and increased surface roughness [235]. The deposition of GO onto zirconia caused an increased hydrophobic tendency compared with pristine zirconia [294]. The GO-coated surface reduced the adhesion of *S. mutans* and promoted their cell behavior. The antibacterial effects of GO coating can be attributed to the physical destruction of bacteria membranes via increased surface hydrophobic interactions and ROS-mediated oxidative stress [295, 296]. However, due to the relatively low hydrophilicity of GO compared to zirconia, further research is required to balance the surface hydrophilicity of GO to facilitate cell behavior. In addition, an excessively thick GO layer reduces the esthetic value of zirconia; thus, it is crucial to determine the minimum coating thickness required.

## Strategies to enhance PEEK implant success

**Fig. 11:**
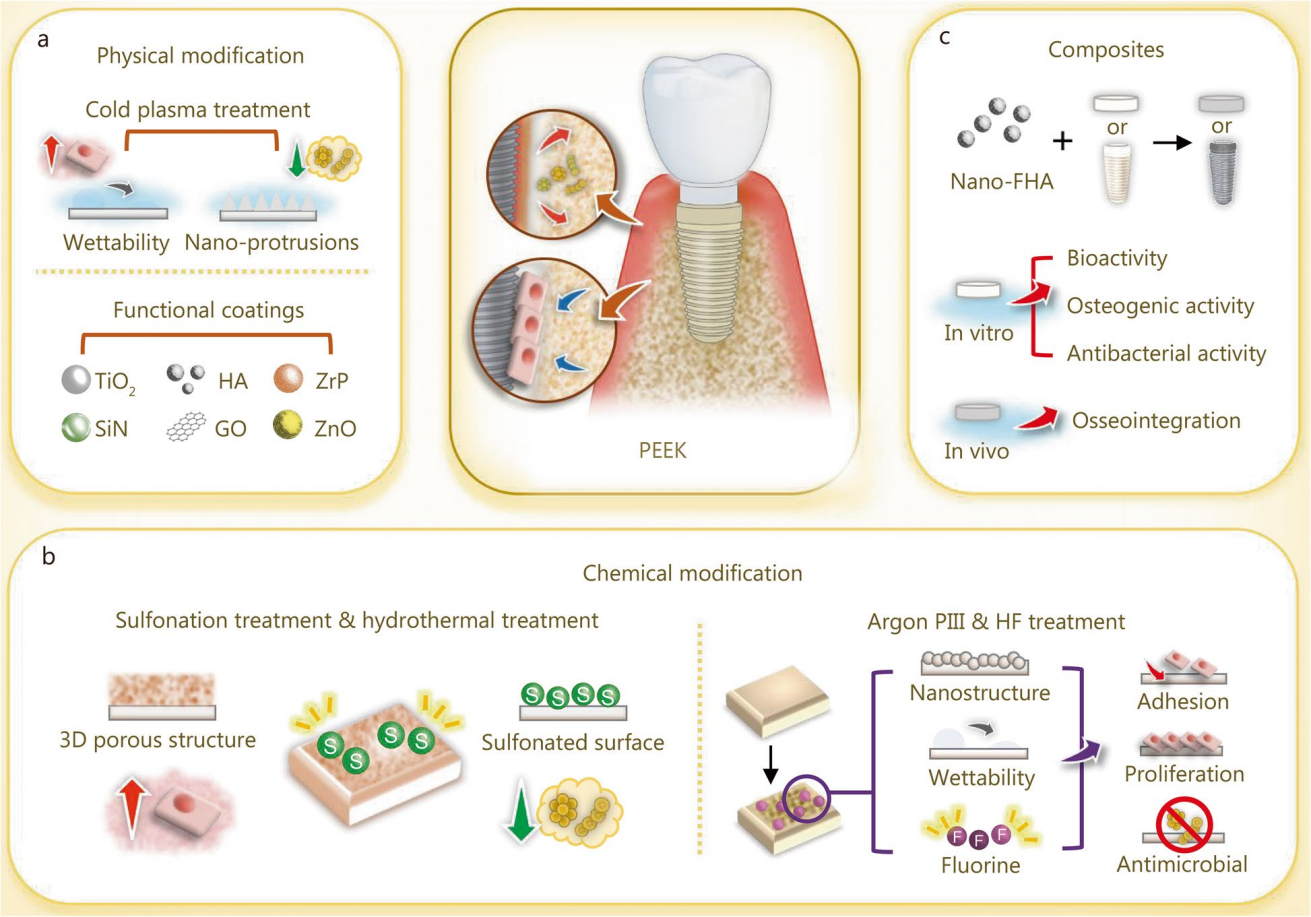
Biomaterials and modification strategies for PEEK dental implant to enhance the osteogenic activities and antibacterial properties. **a** Physical modification techniques. **b** Chemical modification techniques. **c** Composites. PEEK polyether-ether-ketone, TiO_2_ titanium dioxide, HA hydroxyapatite, ZrP zirconium phosphate, SiN silicon nitride, GO graphene oxide, ZnO zinc oxide, HF hydrofluoric acid

PEEK is a member of the polyaryletherketone family, which is characterized by an aromatic backbone that combines ketone and ether functional groups between the aryl rings [33]. PEEK exhibits satisfactory chemical resistance, esthetics, biocompatibility, and bone-like elasticity (3–4 GPa). Consequently, PEEK has emerged as an alternative candidate for titanium-based dental implants [297–300]. Despite the increasing use of PEEK in dental implant applications, its long-term efficiency still needs to be determined. Unmodified PEEK is inherently hydrophobic (water contact angle of 80–90°) and bio-inert [301, 302]. Therefore, various modification strategies have been developed to enhance the biological activity of PEEK implants (Table 7 [99, 303–314], Fig. 11a-c).

**Table 7:** Summary of the PEEK surface modification strategies and the corresponding antibacterial effects

Modification methods	Surface properties	Antibacterial properties	Cell response to the surface	In vivo study	References
Plasma treatment	Hydrophilic surface with nano-protrusions and positively charged functional groups	Inhibited adhesion and growth of *S. mutans* and *S. aureus*	Enhanced osteogenic activity	-	[303]
Acid etching	Porous structure	-	Increased mineralization and osteogenic differentiation	Rat model: increased implant osseointegration	[304]
Accelerated neutral atom beam	Increased roughness, hydrophilicity, and surface-active groups	-	Enhanced cell adhesion, proliferation, and osteogenesis related gene expression	Sheep model: enhanced bone ingrowth	[305–307]
Coating with GO nano-sheets	Cotton-like morphology with enhanced surface hydrophilicity	Inhibitory effect on *P. gingivalis* and *S. mutans*, prevent biofilm formation	Good cytocompatibility and enhanced osteogenic differentiation	-	[308]
Coating with GO and ZnO nanoparticles	ZnO crystals agglomerated into petal lamellar GO	Inhibitory effect on *P. gingivalis* and *S. mutans*	Biocompatible to L929 cells	-	[309]
UV radiation grafting	Changed surface chemistry, enhanced hydrophilicity	-	Enhanced adhesion, proliferation, and osteogenic differentiation	Rabbit model: improved bone-implant contact	[310, 311]
Sulfonation treatment	Three-dimensional porous network with enhanced surface hydrophobicity	Resistance against *S. aureus* and *E. coli*	Enhanced proliferation and osteogenic differentiation	Rat model: enhanced osseointegration and antibacterial resistance	[312, 313]
Plasma immersion ion implantation followed by fluorination treatment	Changed surface chemistry, enhanced hydrophilicity	Bacteriostatic effect against *P. gingivalis*	Enhanced cell adhesion, proliferation, and alkaline phosphatase activity	Rat model: enhanced osseointegration	[99]
Composite	Enhanced surface roughness and hydrophilicity	Inhibited proliferation of *S. mutans*, prevented biofilm formation	Improved cell adhesion, proliferation, mineralization, and osteogenic differentiation	Dog model: promoted osseointegration	[314]

*GO* graphene oxide, *ZnO* zinc oxide, *UV* ultraviolet, *S. mutans Streptococcus mutans, S. aureus Staphylococcus aureus, P. gingivalis Porphyromonas gingivalis, E. coli Escherichia coli*

### Physical surface modification methods

Commonly used physical surface modification strategies for improving PEEK bioactivity include accelerated neutral atom beam, plasma treatment, and surface coating. Cold plasma treatment is a typical physical method used to improve the biological behavior of PEEK. Three types of cold plasma including argon (Ar) plasma, nitrogen (N_2_), and 90% Ar and 10% N_2_ mixture, have been used to treat PEEK substrates [303]. Among them, the N_2_ cold plasma treated surface showed the strongest osteogenic activity and satisfactory antibacterial activity, and is therefore the most suitable modification method for PEEK application in dental implants. This mechanism can be explained by the enhanced microcosmic morphologies and surface hydrophilicity, along with the changes in chemical compositions caused by cold plasma treatment. The surface of pure PEEK has strong hydrophobicity owing to the large number of benzene rings in its molecular backbones. On the contrary, cold plasma treatment can give the surface enhanced surface hydrophilicity and positively charged nitrogen-containing groups, which effectively promotes adhesion, proliferation, and differentiation of osteoblasts. Furthermore, compared with the smooth PEEK surface, the plasma-treated surface exhibits matrix-arranged nano-protrusions, and the distance between adjacent protrusions is smaller than the size of the bacteria. In addition to the positively charged functional groups, cold plasma treatment can effectively limit bacteria adherence and inhibit bacteria growth.

Another modification strategy for improving the bioactivities of PEEK is to coat the surface with functional substances such as TiO_2_ [315, 316], hydroxyapatite (HA) [317, 318], nano zirconium phosphate [319] or nano silicon nitride [320], Moreover, biomaterials with superior antibacterial activity, such as GO and ZnO, have been used as coatings [308, 309]. These coatings effectively inhibited the growth of dental pathogens in in vitro antibacterial experiments. However, from an esthetic point of view, these materials may be disadvantageous. Additionally, the long-term antibacterial effects and in vivo biological performance of these surfaces need to be further evaluated to verify their effectiveness in preventing and managing peri-implantitis.

### Chemical surface modification methods

Chemical surface modification mainly refers to the immobilization of bioactive substances on the surface of PEEK through chemical bonds, to increase the binding strength between functional groups and the substrate. UV radiation grafting is a common method that endows the surface with enhanced osteogenic activity by destroying the carbonyl chemical bonds in the molecular backbone, generating free radicals, and inducing the polymerization of bioactive alkene monomers [310, 311]. However, due to the complexities of the oral microenvironment, materials with single osteogenic properties do not meet the requirements for successful long-term implantation.

To enhance the antibacterial activity, sulfonation treatment was applied to the PEEK surface, and then hydrothermal treatment was performed to remove the residual sulfur-compounds, and different sulfur concentrations were obtained by controlling the treatment temperature [312]. The sulfonated PEEK has a three-dimensional-porous-network structure which is conducive to the proliferation and differentiation of rat bone marrow mesenchymal stem cells. The sulfonated surfaces also exhibit good resistance to *S. aureus* and *E. coli*, and the antibacterial activity is positively correlated with the sulfur content [312]. However, abundant studies have demonstrated that excessive sulfur functional groups have negative effects on human cells [321–323]. The sulfur released by high-sulfur samples might create an acidic environment around the implant, thereby hindering the growth of cells to a certain extent, so a proper balance should be achieved by optimizing the sulfur concentration. In vitro and in vivo experiments indicated that the samples with excessive sulfur removed by hydrothermal treatment showed better osseointegration. Although the samples with higher sulfur content had good antibacterial properties, their harmful effects, including inferior cytocompatibility and attenuated osteogenic ability, cannot be overlooked. In contrast, lower sulfur concentrations resulted in reduced antibacterial effects but increased bone remodeling due to the reduced sulfur release. These results indicate that sulfur concentration must be optimized to achieve the desired antibacterial ability, biocompatibility, and osseointegration.

Fluorine is a microelement essential for human life and is vital for bone growth and the maintenance of physiological functions. Some studies have shown that appropriate fluorine levels can accelerate osteoblast proliferation and differentiation [324–326]. Additionally, fluorine can inhibit bacterial activity, for example, the use of sodium fluoride to prevent caries by inhibiting *streptococcus* [327]. Therefore, fluorination has been used to modify the PEEK surface. Chen et al. [99] prepared fluorinated PEEK via argon plasma immersion ion implantation (PIII), followed by hydrofluoric acid treatment to enhance the antibacterial and osteogenic properties of PEEK. PIII can cause polymer chain scission and provide active sites for interaction with fluorinate ions; hence, argon PIII plays an important role in introducing fluorine to improve the bioactivity of the PEEK substrate. Fluorinated PEEK exhibited a superior bacteriostatic effect against *P. gingivalis* compared with pristine PEEK. Moreover, the fluorine-containing nanostructured surfaces effectively enhanced the proliferation and differentiation of rBMSCs. In vivo, evaluation using micro-CT, sequential fluorescent labeling, and methylene-fuchsin staining revealed that the samples prepared through argon PIII followed by hydrofluoric acid treatment displayed enhanced osseointegration.

### Composites

**Fig. 12:**
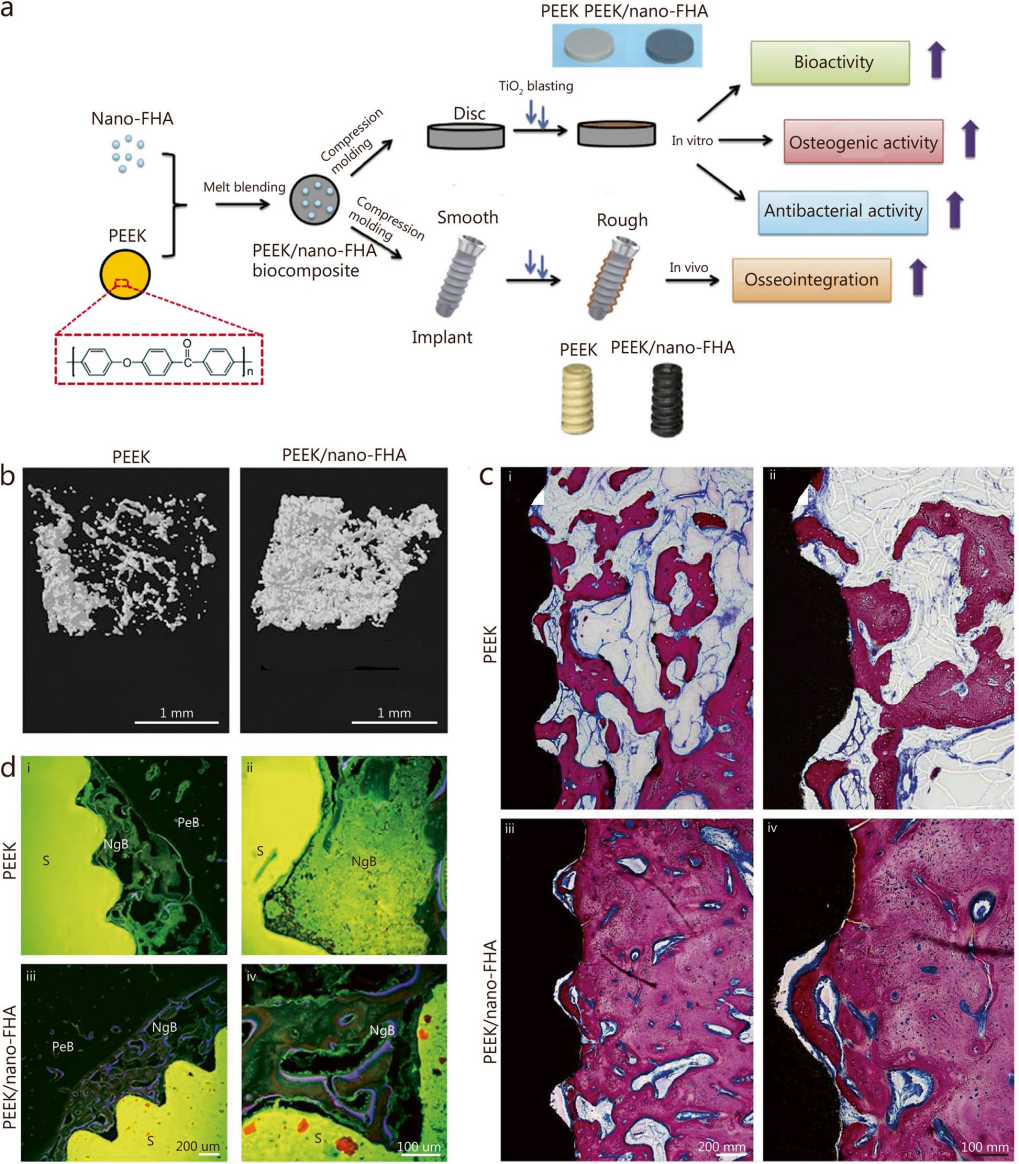
The preparation and in vivo assessments of osseointegration of PEEK/nano-FHA implants. **a** Schematic diagram of the preparation and evaluation of PEEK/nano-FHA composite samples. **b** Micro-CT three-dimensional reconstruction models showing the regenerated bone of about 0.5 mm width ring around PEEK and PEEK/nano-FHA implants surface at 8 weeks. **c** Histotomy of bone contact immunostained by toluidine blue-fuchsine at 8 weeks of bare PEEK (**i-ii**) and PEEK/nano-FHA implants (**iii-iv**) postoperatively. (**ii**) and (**iv**) refer to the higher-magnification images of (**i**) and (**iii**), respectively. The dark red area represents the newly formed bone, and the dark black area represents the PEEK-based implant. White scale: 200 μm, black scale: 100 μm; **d** In histological analysis, new bone formation around bare PEEK (**i-ii**) and PEEK/nano-FHA implants (**iii-iv**) were detected by bone labeling (calcein, calcein blue, and tetracycline). (**ii**) and (**iv**) refer to the higher-magnification images of (**i**) and (**iii**), respectively [314]. Copyright 2014, Elsevier. S sample, NgB newly grown bone deposition and remodeling zone, PeB pre-existing bone tissue zone

Fluorohydroxyapatite (FHA) is a highly bioactive CaP widely used in tissue engineering because of its chemical and crystallographic similarities to natural apatite in natural bone tissues. Unlike pure HA, FHA exhibits much higher physicochemical stability and osteogenic properties [328]. Moreover, the fluorine ions released from massive FHA can be considered antimicrobial drugs, as they can prevent the growth of oral bacteria and the development of biofilms [329]. Therefore, nano-FHA has been used as a nano-reinforcement material to prepare PEEK-based composites for dental applications. Wang et al. [314] developed a PEEK/nano-FHA composite with a rough surface morphology as a dental implant material (Fig. 12a). This composite not only inhibited the attachment and colonization of *S. mutans* but also prevented the formation of biofilm. Because fluoride can affect the activities of the glycolytic enzyme enolase, proton-extruding ATPase, and biofilm-associated enzymes, thereby interfering with bacterial metabolism and dental biofilm acidogenicity [329]. Furthermore, the addition of nano-FHA improved the biocompatibility in vitro and promoted osseointegration in vivo. As shown in Fig. 12b-d, more bone deposition and remodeling were observed around PEEK/nano-FHA implants, suggesting a higher degree of bone regeneration than that of pure PEEK implants. This study paves the way for the utilization of PEEK-based composite as a dental implant material in challenging applications and the improvement of antibacterial activity as well as osteogenic properties.

## Clinical applications of surface-modified dental implants

Although laboratory research on antibacterial surfaces has made great progress, there are few reports on clinical studies. A total of 133 studies were included in this review, and only 3 of which possessed clinical performance. Since zirconia surface requires some special modification methods and PEEK dental implants have not yet entered the market, there are very few clinical studies regarding antibacterial surfaces using zirconia or PEEK as the substrate. Most of the proposed studies mainly focus on Ti and its alloys as the substrate.

Predominantly, the published clinical studies on modification strategies against peri-implantitis involved surface coating with anatase TiO_2_ [129] or antibacterial agents [105, 139] (Table 8). The formation of anatase coating on commercial Ti6Al4V alloy has shown a remarkable inhibitory effect on bacterial attachment and biofilm formation. Upon performing a clinical study of 8 subjects who wore anatase-coated discs consecutively for 24 h without any oral hygiene procedure, it was found that anatase coating formed by anodization at 120 V on Ti6Al4V significantly inhibited biofilm formation [129]. For antibacterial agents, the internal coating of the dental implant with an alcoholic solution containing polysiloxane oligomers and 1% chlorhexidine gluconate was effective in preventing bacterial infection and affecting the microbial species of the pathogens responsible for peri-implantitis. A total of 15 healthy patients were scheduled to receive either bilateral fixed prostheses or crown restorations supported by an implant fixture. After 4-month of implantation, no adverse reactions or implant failure were reported, all the experimental sites exhibited good soft tissue healing, and no local evidence of inflammation was observed [139]. Another clinical study evaluated the durable and renewable antibacterial effect of N-halamine polymeric coating on Ti substrates. Eight subjects were selected for a 4-week experiment, and 3 subjects were selected for a 12-week experiment. For each subject, the coated and uncoated Ti disks were bonded to the middle-third of the buccal surfaces of the upper and lower first molars. The polymeric coatings can effectively inhibit biofilm formation in the human oral cavity due to the gradually dissociated active halogen. After use, they can be washed and rechlorinated with NaOCl solution, making the active halogen easy to regenerate, thus providing long-term prevention and treatment effects [105].

**Table 8:** Clinical applications regarding biomaterials and surface modification strategies for dental peri-implantitis management

Materials	Sample quality	Setting area	Subjects	Follow-up	Main result	References
Anatase TiO_2_ coating on Ti6Al4V discs	48 (8 C/16T)	Oral cavity	8 (3 M/5F), between 20 and 25 years	24 h	Inhibit bacterial attachment and biofilm formation	[129]
Coating the internal part of the Ti dental implant with chlorhexidine gluconate	60 (30 C/30T)	Oral cavity (implants were placed 2 mm under the crestal bone level)	15 (6 M/9F), between 45 and 61 years	6 months	No adverse effects, implant failure, or local inflammation were observed, all experimental sites showed good soft tissue healing	[139]
N-halamine polymeric-coated Ti disks	44 (22 C/22T)	Oral cavity (middle-third of buccal surfaces of upper and lower first molars)	11	4 or 12 weeks	Inhibit biofilm formation, the antibacterial property can be further enhanced by rechlorination	[105]

*TiO*_*2*_ titanium dioxide, *Ti* titanium, *C* control, *T* test, *M* male, *F* female

Although clinical studies have shown positive results, no product has been clinically validated to date. The gap between the number of laboratory research and commercially available products indicates that investigations still encounter some significant hurdles before moving forward to a clinical setting. Indeed, the low clinical translatability of laboratory findings can be attributed to unknown impacts on biosafety and functionality. The main difficulties are as follows. (1) The long-term in vivo performance of antibacterial dental implants should be evaluated for many years to ensure biosafety and efficacy. (2) Current research focuses on the evaluation of antibacterial effects but neglects the overall physiological effects of therapy, such as neovascularization or immune systems. (3) Bacteria may also evolve resistance mechanisms to non-antibiotic therapeutics. Therefore, further studies are urgently required before entering the clinical stage. Given the above obstacles, the next research focus should be to evaluate long-term data of 1–2 years or even 10 years after implantation using suitable animal modes. Besides, it is necessary to evaluate the underlying mechanism of immune cells’ response to the modified dental implants. Moreover, researchers also need to carefully study the bacterial resistance to these novel antibacterial surfaces.

## Conclusions and perspectives

**Fig. 13:**
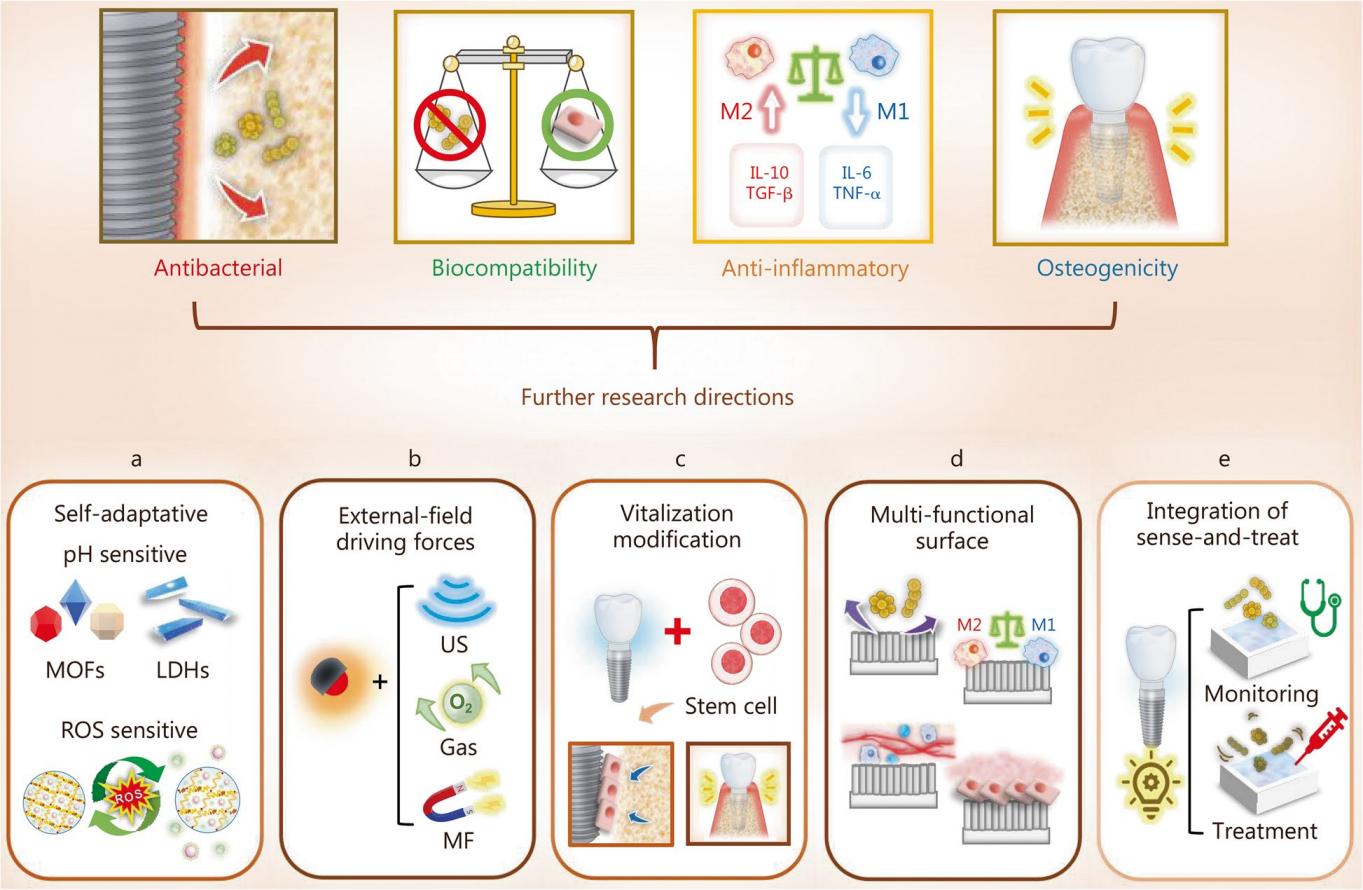
The further research directions of dental implants. **a** Developing self-adaptative antibacterial coatings that function according to the microenvironment. **b** Developing intelligent antibacterial surfaces that can be activated by multiple external-field driving drivers. **c** Developing lively surface to promote osseointegration by using MSCs-based therapy. **d** Developing multi-functional surfaces. Combination of the design rules of osteogenesis-angiogenesis regulation and osteoimmunomodulation with existing antibacterial strategies. **e** Developing versatile surfaces for integrated diagnosis and treatment. M1 M1 type macrophage, M2 M2 type macrophage, IL-10 interleukin-10, TGF-β transforming growth factor-β, IL-6 interleukin-6, TNF-α tumor necrosis factor-α, MOFs metal-organic frameworks, LDHs layered double hydroxides, ROS reactive oxygen species, US ultrasonic, MF magnetic field

Dental implants are permanent replacements for tooth loss, and their long-term functions antibacterial and osteogenic are of great significance for the treatment of peri-implantitis. Based on the clinical treatment requirements, the proposed studies are grouped into the following categories. (1) Application of biomaterials with intrinsic antibacterial properties as the dental implant. Ti-Cu alloys can sustainably release Cu ions to inhibit bone resorption caused by bacterial infection and maintain the microflora balance so that to fulfill the long-term antibacterial demands. (2) Application of novel modification strategies to endow dental implants with long-term or stimuli-responsive antibacterial protection to cover the healing period. N-halamine polymeric coating can persistently inhibit bacterial adhesion and biofilm formation, which can be applied as a conventional coating on the surface of a Ti-based dental implant. Moreover, considering the stability and durability issues, the construction of NIR-responsive TiO_2_ biometasurface on the surface of Ti-based implants possessed efficient antibacterial activity under NIR-irradiation and excellent biosafety, providing a new strategy for the treatment of peri-implant infections. (3) Construction of multi-functional surface to simultaneously achieve antibacterial, anti-inflammatory, and osteogenic functions. Since the elimination of biofilm only removes inflammatory stimuli from the surrounding microenvironment but does not completely improve local inflammation, it may weaken the osseointegration at the implant site. Thereby, surface modifications with anti-inflammatory CeO_2_ nanoparticles or bioactive dendrimer poly(amidoamine) are of great significance for regulating the immune microenvironment and promoting osseointegration. However, it is still challenging to give implants long-term antibacterial function while preserving good biocompatibility and osteogenic activity. In our opinion, the future research directions in this field are summarized as follows (Fig. 13).

Developing self-adaptative antibacterial coatings that function according to the microenvironment (Fig. 13a). Precise and controllable release of antibacterial agents from dental implant surfaces remains a significant clinical challenge. Since bacterial infection can induce an acidic microenvironment (pH 4.5–6.5), which can be considered as a potential stimulus to activate antibacterial actions. Thereby, a series of pH-responsive materials, such as metal-organic frameworks or layered double hydroxides, can be used as delivery vehicles or functional components to construct antibacterial surface coatings that are responsive to the acidic microenvironment [330, 331]. Besides, the excessive production of ROS at the infection site can also be recognized as a stimulus [332]. Therefore, a range of molecules with ROS-reaction functional groups are used to construct ROS-triggered drug release systems to eliminate bacterial infection and achieve oxidative balance. Additionally, based on the metabolic differences between bacteria and cells, the development of heterojunction surface with self-activation properties is conducive to the electron transfer between bacteria and materials, while simultaneously promoting osteoblastic differentiation, to achieve a balance between antibacterial and osteogenic effects [333].

Developing intelligent antibacterial surfaces that can be activated by multiple external-field driving drivers (Fig. 13b). Current research focuses on antimicrobial photodynamic therapy (aPDT) for the treatment of peri-implant infections. However, the hypoxic environment deep in the periodontal pocket may limit the clinical application of aPDT [334]. Therefore, the application of novel external-field driving drivers, such as NIR-triggered phototherapies combined with sonodynamic therapy or gas therapy, to initiate antibacterial actions is an ideal solution to this problem [335, 336]. Besides, eradication of bacterial biofilm can be achieved by the combination of a PDT with the magnetic field (MF). Under transverse rotation of MF and NIR irradiation, the photoactive materials loaded with magnetic nanoparticles can provide mechanical force and generate ROS to destroy oral biofilm [337]. Therefore, the design of antibacterial surfaces in the future may focus on intelligent materials with stimuli-responsive properties. Once bacterial infection occurs, they can be activated by a variety of external-field driving forces. After successfully exerting antibacterial effects, the intelligent surface coating will rapidly decompose without affecting the implant surface morphology or subsequent osseointegration.

Developing active surface to promote osseointegration (Fig. 13c). There is a discrepancy between in vitro and in vivo results for conventional osteogenic biomaterials, with materials that induce bone formation in vitro potentially having poorer effects in vivo. MSCs-based therapy represents a promising alternative to bone graft in the treatment of peri-implant defects. Numerous preclinical studies have demonstrated the treatment outcomes of MSCs-based therapy, including direct injection of MSCs or surface modification with MSCs [338, 339]. Besides, stem cell exosomes can be recognized as a novel cell therapy with lower safety risks and higher therapeutic effects, which effectively alleviates inflammation and promotes targeted tissue regeneration [340, 341]. However, its therapeutic safety and clinical effects in humans remain to be investigated.

Developing multi-functional surfaces (Fig. 13d). Combination of the design rules of osteogenesis-angiogenesis regulation and osteoimmunomodulation with the existing antibacterial strategies is beneficial to reduce the risk of peri-implantitis. Since bone metabolism and regeneration are closely related to the nervous system and immune response [342, 343], and a large number of studies have demonstrated that engineered biomaterials have a positive effect on bone regeneration by promoting neovascularization and M2 macrophage polarization [344, 345]. For example, TiO_2_ nanotubes with appropriate diameters can effectively down-regulate pro-inflammation genes and cytokines, up-regulate anti-inflammation genes and cytokines, and promote angiogenesis, so that facilitate osseointegration of peri-implant during implantation [346]. Meanwhile, TiO_2_ nanotubes can also reduce the adhesion of P. gingivalis to prevent the risk of peri-implantitis [131]. Therefore, how to organically integrate these biological functions into the dental implant surface to construct a multi-functional surface through reasonable design can be considered as a potential development direction.

Developing versatile surface for integrated diagnosis and treatment (Fig. 13e). The oral microenvironment contains a complex microbiome. Due to reduced gingival fibrous arrangement and vascular supply, the peri-implant interface is less effective in resisting bacterial infections than natural teeth, which makes it more vulnerable to peri-implant infections and future bone loss around the implants. To further promote the probability of long-term clinical implant success, the construction of intelligent implants is of great significance in the management of peri-implantitis and can be considered a promising direction in implant design [347]. To this end, a platform for integrated diagnosis and treatment can be incorporated into dental implants to diagnose and treat bacterial biofilm infections [348]. This can be achieved through the introduction of sensing and imaging techniques into the implant to allow real-time monitoring and assessment by sensing variations in the oral environment (e.g., pH, temperature, and bacterial metabolites) to reflect the colony units and infection severity [349–351]. Once abnormalities are identified, the infection data will interact with the terminal device through advanced wireless technology to activate the drug management system for intelligent drug release. The concept of “sense-and-treat” can be considered a promising approach that can be incorporated into the design of dental implants that both sense attached bacteria and provide antibacterial performance.

## Data Availability

The data and materials used during the current review are all available in this review.

## Abbreviations

ALD: Atomic layer deposition
AMPs: Antimicrobial peptides
BMSCs: Bone marrow mesenchymal stem cells
CAT: Catalase
CaP: Calcium phosphate
DEP: Diethyl phosphite
DMADDM: Dimethylaminododecyl methacrylate
G: Graphene
GO: Graphene oxide
HA: Hydroxyapatite
MAO: Macro-arc oxidation
NIR: Near-infrared ray
PAMAM: Poly(amidoamine)
PDT: Photodynamic therapy
PEEK: Polyether-ether-ketone
PEG: Poly(ethylene glycol)
rGO: Reduced graphene oxide
ROS: Reactive oxygen species
SLA: Sandblasting and acid etching
SOD: Superoxide dismutase
Ti-Cu: Titanium-copper
TiN: Titanium nitride
TNF-α: Tumor necrosis factor-α
ZnO NRS: ZnO nanorods and ZnO nanospheres
